# Supplementary material for: Neuropsychiatric safety of varenicline in the general and COPD population with and without psychiatric disorders: a retrospective cohort study in a real-world setting
Source: BMJ Open. 2021 May 25;11(5):e042417. doi: 10.1136/bmjopen-2020-042417 (PMC8154988; doi:10.1136/bmjopen-2020-042417)
Supplement: Supplementary data [file bmjopen-2020-042417supp001.pdf]

Supplementary Materials

Table S1. List of ATC codes used for identification of related diseases and outcome events.

Variables	ATC codes*	Explanation
COPD	R03	- drugs for obstructive airway diseases;
General psychiatric disorders	N02, N03, N04, N05, N06	- analgesics;<br>- antiepileptics;<br>- anti-Parkinson drugs;<br>- psycholeptics;<br>- psychoanaleptics;
Depression	N06A, N06CA,	- antidepressants;<br>- antidepressants in combination with psycholeptics;
Anxiety	N05B	- anxiolytics;
Insomnia	N05C	- hypnotics and sedatives
Heart failure	C01AA, C03C	- digitalis glycosides<br>- high-ceiling diuretics
Ischemic heart disease	C01DA	- organic nitrates
Hypertension	C02, C03 (except C03C), C07, C08, C09	- antihypertensives<br>- diuretics (except high-ceiling diuretics)<br>- beta blocking agents<br>- calcium channel blockers<br>- agents acting on the renin-angiotensin system
Cancers	L01	- antineoplastic agents
Diabetes mellitus	A10	- drugs used in diabetes
Osteoporosis	M05B	- drugs affecting bone structure and mineralization
Peptic ulcer and GERD	A02B	- drugs for peptic ulcer and gastro-oesophageal reflux disease (gord)
Rheumatic arthritis	M01, M02	- antiinflammatory and antirheumatic products<br>- topical products for joint and muscular pain

Thyroid disorders	H03	- thyroid therapy
Anemia	B03	- antianemic preparations
Glaucoma	S01E	- antiglaucoma preparations and miotics
Gout	M04	- antigout preparations
Allergic rhinitis	R01AD	- corticosteroids

ATC: anatomical therapeutic chemical; COPD: chronic obstructive pulmonary disease; GERD: gastroesophageal reflux disease; *The very specific medication lists under each ATC codes level could be checked in WHO ATC system: https://www.whocc.no/atc_ddd_index/.

Table S2. Subgroup analysis: incidence of neuropsychiatric adverse events (NPAEs) and association with varenicline compared with NRT within follow up of 24 weeks stratified by age and gender.

Subgroups	General populations (N=9077)				COPD populations (N=1598)			
	Psychiatric cohort Varenicline(n=1427) vs NRT(1200)		Non-psychiatric cohort Varenicline(n=4480) vs NRT(n=1970)		Psychiatric cohort Varenicline(n=327) vs NRT(322)		Non-psychiatric cohort Varenicline(n=608) vs NRT(n=341)	
	Crude OR(95% CI)	aOR (95% CI)	Crude OR (95% CI)	aOR (95% CI)	Crude OR (95% CI)	aOR (95% CI)	Crude OR (95% CI)	aOR (95% CI)
Age **								
Age < 40	0.58 [0.32, 1.05]	0.54 [0.28, 1.04]	1.01 [0.61, 1.66]	0.93 [0.54, 1.64]	---	---	---	---
40-65	0.81 [0.65, 1.01]	0.78 [0.63, 0.98]	0.86 [0.71, 1.04]	0.87 [0.72, 1.07]	0.88 [0.56, 1.38]	0.87 [0.54, 1.41]	0.91 [0.57, 1.46]	0.93 [0.57, 1.53]
>65	1.02 [0.66, 1.59]	1.13 [0.70, 1.83]	0.50 [0.33, 0.78]	0.53 [0.34, 0.83]	1.08 [0.50, 2.35]	1.02 [0.43, 2.40]	0.51 [0.25, 1.05]	0.51 [0.24, 1.11]
Gender (n, %) **								
Men	0.87 [0.67, 1.13]	0.89 [0.68, 1.16]	0.71 [0.56, 0.91]	0.78 [0.61, 0.99]	0.95 [0.54, 1.69]	1.24 [0.66, 2.33]	0.81 [0.46, 1.43]	1.00 [0.53, 1.88]
Female	0.73 [0.56, 0.95]	0.74 [0.57, 0.97]	0.89 [0.71, 1.11]	0.91 [0.72, 1.14]	0.78 [0.46, 1.33]	0.77 [0.44, 1.35]	0.73 [0.44, 1.22]	0.76 [0.44, 1.31]

NPAEs: neuropsychiatric adverse events; NRTs: nicotine replacement therapy; OR: odds ratio; CI: confidence interval; aOR: adjusted odds ratio; *adjusted gender, social economic status and comorbidities; **adjusted age, social economic status, comorbidities.

Table S3. Sensitivity analysis: incidence of neuropsychiatric adverse events (NPAEs) and association with varenicline compared with NRT in general population with and without psychiatric disorders within follow up of 12 weeks.

NPAEs	**Psychiatric cohort (N=2627)** Varenicline (n=1427) vs NRTs (n=1200)			**Non-psychiatric cohort (N=6450)** Varenicline (n=4480) vs NRTs (n=1970)		
	Events (n, %)*	Crude OR (95% CI)	Adjusted OR# (95% CI)	Events (n, %)*	Crude OR (95% CI)	Adjusted OR# (95% CI)
**Follow-up of 12 weeks**						
Overall	1062 (74.4): 948 (79.0)	0.77 [0.64, 0.93]	0.78 [0.64, 0.94]	363 (8.1): 223 (11.3)	0.69 [0.58,0.82]	0.74 [0.62, 0.89]
Depression	560 (39.2): 491 (40.9)	0.93[0.80, 1.09]	0.89 [0.76, 1.05]	86 (1.9): 30 (1.5)	1.27 [0.83, 1.92]	1.29 [0.84, 1.97]
Anxiety	367 (25.7): 423 (35.3)	0.64 [0.54, 0.75]	0.63 [0.53, 0.74]	119 (2.7): 61 (3.1)	0.85 (0.62, 1.17)	0.90 [0.65, 1.24]
Insomnia	321 (22.5): 311 (25.9)	0.83 [0.69, 0.99]	0.88 [0.73, 1.05]	82 (1.8): 61 (3.1)	0.58 [0.42, 0.82]	0.62 [0.44, 0.88]

NPAE: neuropsychiatric adverse events; OR: odds ratio; CI: confidence interval; NRTs: nicotine replacement therapy; # adjusted for age, gender, social economic status and related comorbidities. *Varenicline:NRT.

Table S4. Sensitivity analysis: incidence of neuropsychiatric adverse events (NPAEs) and association with compared with NRT in general population within follow up of 24 weeks.

Limitations	General populations (N=9077)					
	Psychiatric cohort varenicline(n=1427) vs NRTs (n=1200)			Non-psychiatric cohort varenicline(n=4480) vs NRTs (n=1970)		
	Events (n, %) #	OR (95% CI)	aOR (95% CI) *	Events (n, %) #	OR (95% CI)	aOR (95% CI) *
**Exclude participants prescribed drugs for any NSP within 1 month before index date**						
Overall	254 (57.6): 157 (60.4)	0.89 [0.65, 1.22]	0.87 [0.63, 1.21]	418 (9.6): 192 (10.4)	0.91 [0.76, 1.10]	0.93 [0.78, 1.12]
Depression	158 (35.8): 82 (31.5)	1.21 [0.88, 1.68]	1.18 [0.84, 1.67]	139 (3.2): 50 (2.7)	1.18 [0.85, 1.64]	1.16 [0.84, 1.62]
Anxiety	78 (17.7): 62 (23.8)	0.69 [0.47, 0.10]	0.68 [0.46, 1.01]	197 (4.5): 85 (4.6)	0.98 [0.76, 1.27]	1.02 [0.78, 1.33]
Insomina	75 (17.0): 48 (18.5)	0.94 [0.74, 1.20]	0.94 [0.62, 1.43]	131 (3.0): 91 (4.9)	0.60 [0.46, 0.79]	0.61 [0.46, 0.80]
**Exclude participants prescribed drugs for depression, anxiety and insomnia within 1 month before index date**						
Overall	314 (48.9): 201 (46.1)	1.12 [0.88, 1.43]	1.05 [0.82, 1.36]	424 (9.6): 203 (10.6)	0.90 [0.75, 1.07]	0.92 [0.77, 1.11]
Depression	191 (29.8): 104 (23.9)	1.35 [1.02, 1.79]	1.25 [0.93, 1.66]	143 (3.3): 52 (2.7)	1.20 [0.87, 1.66]	1.19 [0.86, 1.65]
Anxiety	99 (15.4): 81 (18.6)	0.80 [0.58, 1.10]	0.75 [0.54, 1.05]	199 (4.5): 92 (4.8)	0.94 [0.73, 1.21]	0.98 [0.75, 1.26]
Insomnia	92 (14.3): 63 (14.4)	0.99 [0.70, 1.40]	1.03 [0.72, 1.47]	132 (3.0): 94 (4.9)	0.60 [0.46, 0.78]	0.61 [0.47, 0.81]
**Exclude participants whose study period includes policies changes**						
Overall	797 (76.0): 733 (77.7)	0.91 [0.74, 1.12]	0.86 [0.69, 1.07]	352 (11.1): 205 (12.9)	0.85 [0.70, 1.02]	0.86 [0.71, 1.03]
Depression	468 (44.7): 411 (43.6)	1.04 [0.88, 1.25]	0.98 [0.82, 1.18]	115 (3.6): 45 (2.8)	1.30 [0.91, 1.84]	1.28 [0.90, 1.82]
Anxiety	343 (32.7): 372 (39.4)	0.75 [0.62, 0.90]	0.72 [0.60, 0.87]	169 (5.3): 95 (6.0)	0.89 [0.69, 1.15]	0.91 [0.70, 1.19]
Insomnia	273 (26.0): 280 (29.7)	0.83 [0.69, 1.02]	0.87 [0.71, 1.07]	109 (3.4): 83 (5.2)	0.65 [0.48, 0.87]	0.66 [0.49, 0.88]
**Exclude selection bias for treatments by applying the inverse probability weighted regression**						
Overall	1074 (75.3): 942 (78.5)	0.83 [0.69, 1.00]	0.88 [0.77, 1.00]	469 (10.5): 248 (12.6)	0.81 [0.69, 0.96]	0.89 [0.80, 0.99]

Depression	629 (44.1): 548 (45.7)	0.94 [0.80, 1.09]	0.90 [0.81,1.00]	148 (3.3): 57 (2.9)	1.15 [0.84, 1.56]	1.14 [0.94, 1.39]
Anxiety	456 (32.0): 472 (39.3)	0.72 [0.62, 0.85]	0.71 [0.63, 0.80]	215 (4.8): 110 (5.6)	0.85 [0.67, 1.08]	0.92 [0.78, 1.07]
Insomina	372 (26.1): 352 (29.3)	0.85 [0.72, 1.01]	0.89 [0.79, 1.00]	147 (3.3): 105 (5.3)	0.60 [0.47, 0.78]	0.63 [0.53, 0.75]

NPAEs: neuropsychiatric adverse events; NRTs: nicotine replacement therapy; OR: odds ratio; aOR: adjusted odds ratio; CI: confidence interval; *adjusted age, gender, social economic status and comorbidities. We set the indexdate was within period between July 1st, 2011 and June 30, 2013; Policy change: In the Netherlands, pharmacologic Smoking Cessation Treatments (pSCTs) were reimbursed in 2011. In 2012 the reimbursement was discontinued. As of 2013, pSCTs were again reimbursed, provided they are accompanied by behavioural counselling. #Varenicline: NRT.

Table S5. Sensitivity analysis: incidence of neuropsychiatric adverse events (NPAEs) and association with varenicline compared with NRT in COPD population with and without psychiatric disorders within follow up of 12 weeks.

Outcomes	**Psychiatric cohort (N=649)** Varenicline (n=327) vs NRTs (n=322)			**Non-psychiatric cohort (N=949)** Varenicline (n=608) vs NRTs (n=341)		
	Events (n, %)#	Crude OR (95% CI)	Adjusted OR* (95% CI)	Events (n, %)#	Crude OR (95% CI)	Adjusted OR* (95% CI)
**Follow-up of 12 weeks**						
Overall	260 (79.5): 267 (82.9)	0.80 [0.54, 1.19]	0.89 [0.59, 1.36]	63 (10.4): 45 (13.2)	0.76 [0.51, 1.14]	0.84 [0.54, 1.29]
Depression	134 (41.0): 127 (39.4)	1.07 [0.78, 1.46]	0.94 [0.67, 1.32]	13 (2.1): 5 (1.5)	1.47 [0.52, 4.15]	1.95 [0.65, 5.91]
Anxiety	95 (29.1): 123 (38.2)	0.66 [0.48, 0.92]	0.64 [0.45, 0.90]	16 (2.6): 13 (3.8)	0.68 [0.32, 1.44]	0.68 [0.31, 1.52]
Insomnia	88 (26.9): 114 (35.4)	0.67 [0.48, 0.94]	0.72 [0.51, 1.03]	20 (3.3): 11 (3.2)	1.02 [0.48, 2.16]	1.12 [0.51, 2.46]

NPAE: neuropsychiatric adverse events; OR: odds ratio; CI: confidence interval; NRTs: nicotine replacement therapy; *adjusted for age, gender, social economic status and related comorbidities. #Varenicline:NRT.

Table S6. Sensitivity analysis: incidence of neuropsychiatric adverse events (NPAEs) and association with compared with NRT in COPD population within follow up of 24 weeks.

Limitations	COPD populations					
	Psychiatric cohort (N=649) varenicline(n=327) vs NRTs(322)			Non-psychiatric cohort (N=949) varenicline(608) vs NRTs(341)		
	Events (n, %)[#]	OR (95% CI)	aOR (95% CI)[*]	Events (n, %)[#]	OR (95% CI)	aOR (95% CI)[*]
**Exclude participants prescribed drugs for any NSP within 1 month before index date**						
Overall	54 (60.0): 39 (61.9)	0.92 [0.48, 1.79]	0.85 [0.41, 1.77]	72 (12.1): 40 (12.7)	0.95 [0.63, 1.43]	0.92 [0.60, 1.43]
Depression	29 (32.2): 19 (30.2)	1.10 [0.55, 2.21]	0.88. [0.39, 1.97]	20 (3.4): 10 (3.2)	1.06 [0.49, 2.29]	1.12 [0.50, 2.52]
Anxiety	22 (24.4): 20 (31.7)	0.70 [0.34, 1.42]	0.87 [0.40, 1.90]	38 (6.4): 19 (6.0)	1.06 [0.60, 1.87]	1.10 [0.60, 2.01]
Insomina	15 (16.7): 16 (25.4)	0.59 [0.27, 1.30]	0.60 [0.24, 1.49]	25 (4.2): 16 (5.1)	0.82 [0.43, 1.56]	0.71 [0.36, 1.37]
**Exclude participants prescribed drugs for depression, anxiety and insomnia within 1 month before index date**						
Overall	68 (51.1): 48 (47.5)	1.16 [0.69, 1.94]	1.29 [0.73, 2.26]	73 (12.1): 43 (13.0)	0.92 [0.62, 1.38]	0.93 [0.61, 1.42]
Depression	35 (26.3): 23 (22.8)	1.21 [0.66, 2.22]	1.08 [0.56, 2.08]	21 (3.5): 10 (3.0)	1.16 [0.54, 2.49]	1.33 [0.60, 2.99]
Anxiety	27 (20.3): 23 (22.8)	0.86 [0.46, 1.62]	0.97 [0.49, 1.91]	38 (6.3): 21 (6.4)	0.99 [0.57, 1.72]	1.00 [0.56, 1.79]
Insomnia	21 (15.8): 20 (19.8)	0.76 [0.39, 1.49]	0.87 [0.42, 1.82]	25 (4.2): 17 (5.2)	0.80 [0.43, 1.50]	0.72 [0.37, 1.39]
**Exclude participants whose study period includes policies changes**						
Overall	203 (78.1): 211 (80.2)	0.88 [0.58, 1.34]	0.97 [0.62, 1.51]	63 (14.2): 41 (15.1)	0.93 [0.61, 1.43]	1.00 [0.64, 1.58]
Depression	114 (43.8): 110 (41.8)	1.09 [0.77, 1.54]	0.96 [0.66, 1.40]	18 (4.1): 7 (2.6)	1.60 [0.66, 3.89]	1.73 [0.68, 4.42]
Anxiety	89 (34.2): 109 (41.4)	0.74 [0.52, 1.05]	0.72 [0.49, 1.05]	35 (7.9): 20 (7.4)	1.08 [0.61, 1.91]	1.21 [0.66, 2.24]
Insomnia	81 (31.2): 106 (40.3)	0.67 [0.47, 0.96]	0.75 [0.51, 1.10]	19 (4.3): 15 (5.5)	0.77 [0.38, 0.53]	0.71 [0.34, 1.45]
**Exclude selection bias for treatments by applying the inverse probability weighted regression**						
Overall	258 (78.9): 260 (80.7)	0.89 [0.61, 1.31]	0.99 [0.76, 1.30]	76 (12.5): 53 (15.5)	0.78 [0.53, 1.13]	0.82 [0.63, 1.07]

Depression	146 (44.6): 145 (45.0)	0.99 [0.72, 1.34]	0.90 [0.73, 1.12]	22 (3.6): 10 (2.9)	1.24 [0.58, 2.66]	1.39 [0.84, 2.29]
Anxiety	110 (33.6): 136 (42.2)	0.69 [0.50, 0.95]	0.67 [0.54, 0.84]	38 (6.3): 25 (7.3)	0.84 [0.50, 1.42]	0.87 [0.60, 1.25]
Insomina	102 (31.2): 124 (38.5)	0.72 [0.52, 1.00]	0.86 [0.68, 1.08]	25 (4.1): 21 (6.2)	0.65 [0.36, 1.19]	0.62 [0.41, 0.94]

NPAEs: neuropsychiatric adverse events; NRTs: nicotine replacement therapy; OR: odds ratio; *aOR: adjusted odds ratio; *adjusted age, gender, social economic status and comorbidities. # Varenicline: NRT. We set the indexdate was within period between July 1st, 2011 and June 30, 2013; Policy change: In the Netherlands, pharmacologic Smoking Cessation Treatments (pSCTs) were reimbursed in 2011. In 2012 the reimbursement was discontinued. As of 2013, pSCTs were again reimbursed, provided they are accompanied by behavioural counselling.
