# Supplementary material for: Neuropsychiatric safety of varenicline in the general and COPD population with and without psychiatric disorders: a retrospective cohort study in a real-world setting
Source: BMJ Open. 2021 May 25;11(5):e042417. doi: 10.1136/bmjopen-2020-042417 (PMC8154988; doi:10.1136/bmjopen-2020-042417)
Supplement: Author's manuscript [file bmjopen-2020-042417.draft_revisions.pdf]

BMJ Open

BMJ Open is committed to open peer review. As part of this commitment we make the peer review history of every article we publish publicly available.

When an article is published we post the peer reviewers' comments and the authors' responses online. We also post the versions of the paper that were used during peer review. These are the versions that the peer review comments apply to.

The versions of the paper that follow are the versions that were submitted during the peer review process. They are not the versions of record or the final published versions. They should not be cited or distributed as the published version of this manuscript.

BMJ Open is an open access journal and the full, final, typeset and author-corrected version of record of the manuscript is available on our site with no access controls, subscription charges or pay-per-view fees (http://bmjopen.bmj.com).

If you have any questions on BMJ Open's open peer review process please email info.bmjopen@bmj.com

Neuropsychiatric safety of varenicline in the general and COPD population with and without psychiatric disorders: a retrospective inception cohort study in a real-world setting

Journal:	*BMJ Open*
Manuscript ID	bmjopen-2020-042417
Article Type:	Original research
Date Submitted by the Author:	04-Jul-2020

Complete List of Authors:	Wang, Yuanyuan; University of Groningen, Groningen Research Institute of Pharmacy, Department of PharmacoTherapy, -Epidemiology and -Economics<br>Bos, Jens; University of Groningen Groningen Research Institute of Pharmacy, Department of PharmacoTherapy, -Epidemiology & -Economics<br>Schuiling-Veninga, Catharina; University of Groningen Groningen Research Institute of Pharmacy, Department of PharmacoTherapy, -Epidemiology & -Economics<br>Boezen, H. Marike; University of Groningen, University Medical Center Groningen, Department of Epidemiology; University of Groningen, University medical center Groningen, Groningen Research Institute for Asthma and COPD (GRIAC)<br>van Boven, Job F. M.; University of Groningen, University Medical Center Groningen, Groningen Research Institute for Athma and COPD (GRIAC) ; University of Groningen, University Medical Center Groningen, Department of Clinical Pharmacy & Pharmacology<br>Wilffert, B; University of Groningen Groningen Research Institute of Pharmacy, Department of PharmacoTherapy, -Epidemiology & -Economics; University of Groningen, University Medical center Groningen, Department of Clinical Pharmacy & Pharmacology<br>Hak, Eelko; University of Groningen Groningen Research Institute of Pharmacy, Department of PharmacoTherapy, -Epidemiology & -Economics; University of groningen, University Medical Center Groningen, Deparment of Epidemiology

Keywords:	Depression & mood disorders < PSYCHIATRY, Anxiety disorders < PSYCHIATRY, EPIDEMIOLOGY

SCHOLARONE™
Manuscripts

I, the Submitting Author has the right to grant and does grant on behalf of all authors of the Work (as defined in the below author licence), an exclusive licence and/or a non-exclusive licence for contributions from authors who are: i) UK Crown employees; ii) where BMJ has agreed a CC-BY licence shall apply, and/or iii) in accordance with the terms applicable for US Federal Government officers or employees acting as part of their official duties; on a worldwide, perpetual, irrevocable, royalty-free basis to BMJ Publishing Group Ltd ("BMJ") its licensees and where the relevant Journal is co-owned by BMJ to the co-owners of the Journal, to publish the Work in this journal and any other BMJ products and to exploit all rights, as set out in our licence.

The Submitting Author accepts and understands that any supply made under these terms is made by BMJ to the Submitting Author unless you are acting as an employee on behalf of your employer or a postgraduate student of an affiliated institution which is paying any applicable article publishing charge ("APC") for Open Access articles. Where the Submitting Author wishes to make the Work available on an Open Access basis (and intends to pay the relevant APC), the terms of reuse of such Open Access shall be governed by a Creative Commons licence – details of these licences and which Creative Commons licence will apply to this Work are set out in our licence referred to above.

Other than as permitted in any relevant BMJ Author's Self Archiving Policies, I confirm this Work has not been accepted for publication elsewhere, is not being considered for publication elsewhere and does not duplicate material already published. I confirm all authors consent to publication of this Work and authorise the granting of this licence.

For peer review only

Neuropsychiatric safety of varenicline in the general and COPD population with and without psychiatric disorders: a retrospective inception cohort study in a real-world setting

Yuanyuan Wang[1], Jens H. Bos[1], Catharina C.M. Schuiling-Veninga[1], H. Marike Boezen[2,3], Job F.M. van Boven[3,4], Bob Wilffert[1,4], Eelko Hak[1,2]

[1]University of Groningen, Groningen Research Institute of Pharmacy, Department of PharmacoTherapy, -Epidemiology & -Economics, Groningen, The Netherlands

[2]University of Groningen, University Medical Center Groningen, Department of Epidemiology, Groningen, The Netherlands.

[3]University of Groningen, University Medical Center Groningen, Groningen Research Institute for Asthma and COPD (GRIAC), Groningen, Netherlands.

[4]University of Groningen, University Medical Center Groningen, Department of Clinical Pharmacy & Pharmacology, Groningen, The Netherlands.

[*]**Corresponding author**: Dr. Yuanyuan Wang,

Department of PharmacoTherapy, -Epidemiology & -Economics,

Groningen Research Institute of Pharmacy, University of Groningen.

P.O. BOX.196, 9700 AD Groningen, The Netherlands.

Tel. +31 (0) 50-363-9163; Fax: +31 (0) 50-363-2772.

Email: yuanyuan.wang@rug.nl; yuanyuanwang.research@gmail.com;

Keywords: smoking cessation; varenicline; nicotine replacement therapy; adverse drug events;

Text word count: 3694

Abstract word: 299

For peer review only - http://bmjopen.bmj.com/site/about/guidelines.xhtml

Abstract

Objectives: Although varenicline is effective for smoking cessation, evidence on its real-world neuropsychiatric safety is inconsistent, notably for high-risk populations. This study aims to evaluate the real-world association between varenicline and neuropsychiatric adverse events (NPAEs) in the general and COPD population with and without psychiatric disorders.

Design: A retrospective inception cohort study.

Setting: This study was based on a large prescription database which includes 70 community pharmacies covering a representative population of approximately 700,000 persons in the Netherlands.

Participants: New users of varenicline or nicotine replacement therapy (NRT) among all adults and COPD patients (≥ 40 years) identified by prescriptions of related drugs.

Outcome measures: The incidence of any drug-treated NPAEs including depression, anxiety and insomnia within 24 weeks after treatment initiation.

Results: In the general population without psychiatric disorders, the incidence of total NPAEs in varenicline (4480) and NRT (1970) groups was 10.5% and 12.6%, respectively (adjusted OR [aOR] 0.85, 95% CI 0.72 to 1.00). In the general population with psychiatric disorders, the incidence of total NPAEs was much higher, 75.3% and 78.5% for varenicline (1427) and NRT (1200) groups, respectively (aOR 0.82, 95% CI 0.68 to 0.99). In the COPD population (1958), there were no differences in the incidence of NPAEs between comparison groups in both the psychiatric cohort (aOR 0.97, 95% CI 0.66 to 1.44) and the non-psychiatric cohort (aOR 0.81, 95% CI 0.54 to 1.20). Results from subgroup or sensitivity analyses also did not reveal increased risks of NPAEs associated with varenicline.

Conclusions: Despite potential healthy user bias, varenicline is unlikely to substantially increase the risk of NPAEs in both the general and COPD population compared with NRT, irrespective of the presence of psychiatric disorders. Our results from a large real-world study may provide reassurance for patients and physicians about the neuropsychiatric safety of varenicline.

For peer review only - http://bmjopen.bmj.com/site/about/guidelines.xhtml

Strengths and Limitations of This Study

- The use of large real-world population data makes the findings from this study more applicable to daily clinical practice.
- The risk of neuropsychiatric adverse events associated with varenicline were explored in both the general population and potential COPD patients.
- Subgroup and sensitivity analysis were performed to confirm the robustness and generalizability of the study findings.
- Despite the control for potential measured confounding and stratification according to risk groups, unmeasured healthy user bias may still be present, but is unlikely to mask substantial risks of varenicline.
- Behavioral changes like self-harm or suicide could not be evaluated in this study.

For peer review only

For peer review only - http://bmjopen.bmj.com/site/about/guidelines.xhtml

Introduction

Tobacco smoking is the leading preventable risk factor for a range of physical and mental illnesses,[1-4] which poses enormous threats to global public health.[5] Although average global smoking rates have declined since 1990 through tobacco control policies,[6] the actual number of smokers and disease burden related to smoking continues to increase owing to population growth.[7] More than 8 million people are killed by tobacco use each year.[8] Therefore, more intensified efforts are needed to fight this deadly epidemic. Smoking cessation strategies as key interventions to prevent smoking-related diseases are therefore urgently needed.[9] Varenicline was the first non-nicotine pharmacotherapy for smoking cessation and has greater efficacy than bupropion, single form nicotine replacement therapy (NRT) or placebo.[10,11] However, substantial concerns regarding its neuropsychiatric safety (e.g. suicidal thoughts, aggressive behavior) have been raised since its approval in the United States in 2006.[12] Therefore, after the first safety communication and public health advisory in 2008, the FDA released a black box warning on July 1, 2009.[13] Of note, these reports could not establish the causality because of a lack of control or comparator. Afterwards, many randomized controlled trials (RCTs) were conducted to evaluate the possible risk of neuropsychiatric adverse events (NPAEs). Notably, pooled evidence of these RCTs did not indicate an association between varenicline and NPAEs.[14] Neither did the Evaluating Adverse Events in a Global Smoking Cessation Study (EAGLES) show a significant increase in NPAEs with varenicline relative to NRT or placebo.[10]

Although the FDA's Black Box warning in varenicline's product labelling was already removed in December 2016, there are still concerns among patients and physicians about its neuropsychiatric safety resulting from the limited external validity of existing evidence from RCTs.[15] Indeed, due to the strict inclusion and exclusion criteria of RCTs, trial participants were generally healthy. Special risk populations with increased smoking prevalence, such as those with COPD and psychiatric disorders, were usually excluded.[16] Importantly, COPD patients are older and suffer from many comorbidities making these patients more susceptible to drug-drug interactions potentially leading to related adverse drug events (ADEs).[17] Similarly, it has been reported that individuals with psychiatric disorders are prone to experience relapse of psychiatric symptoms.[18,19] Varenicline safety in these specific populations

For peer review only - http://bmjopen.bmj.com/site/about/guidelines.xhtml

is not well explored. Only few studies assessed the neuropsychiatric safety of varenicline in patients with COPD or psychiatric disorders,[10,20-22] yet results were inconsistent and related evidence from real-world setting is still lacking.

We therefore conducted this cohort study based on real-life data to assess the risk of NPAEs in starters with varenicline versus NRT starters in both the general and the COPD population with and without psychiatric disorders.

Methods

Study design and setting

We conducted a retrospective inception cohort study based on the University of Groningen pharmacy dispensing database IADB.nl (http://www.iadb.nl/) which has been widely used for various drug utilization studies.[23] IADB.nl contains information of prescribed medications from 70 community pharmacies covering a representative population of approximately 700,000 persons of the Netherlands, regardless of insurance type. It provides both patient information (e.g. date of birth, gender) and complete prescription records including the date of dispensing, quantity dispensed, dose regimen, the number of days the drug will be used, and the related Anatomical Therapeutic Chemical (ATC) code. The ATC code is a unique code assigned to a medicine according to the organ or system it works on and how it works. The ATC classification system proposed by the World Health Organization is a widely accepted drug classification scheme.

Study population

We included adult patients (>18 years) who started with varenicline or NRT. The individuals only prescribed varenicline (ATC code: N07BA03) or NRT (N07BA01) were included as anti-smoking drug users from the general population. Individuals that were prescribed drugs for obstructive airway disease (R03) at least 3 times within 1 year since 1st prescription after the age of 40 years were defined as COPD patients, COPD patients who were prescribed varenicline or NRT were included as COPD anti-smoking drug users.

For peer review only - http://bmjopen.bmj.com/site/about/guidelines.xhtml

For both the general and COPD population with anti-smoking drug use, the first prescription date of varenicline and NRT was set as entry date (index date) of participants for exposure and control groups, respectively. To exclude the influence of other smoking cessation drugs on NPAEs, those who were prescribed other smoking cessation drugs including bupropion (N06AX12), nortriptyline (N06AA10) and cytisine (N07BA04) rather than the studied drugs (varenicline, NRT) within 180 days before or 180 days after index date were excluded. In order to get enough prescription records to define comorbidities and NPAEs, those who were registered in IADB.nl less than 6 months before or after index date were also excluded. For individuals who were prescribed both varenicline and NRT and met criteria of both groups (see Figure 1), we allocated the study subject to the group with the first index date according to the intention-to-treat principle.

In both the general and the COPD population, we classified the individuals into a psychiatric cohort and non-psychiatric cohort according to the presence of psychiatric disorders defined by the prescription of two or more drugs from the neurological ATC group, i.e. N02, N03, N04, N05, N06 within 6 months before index date. In all, our study population covered four separate cohorts (1. General population with psychiatric disorders, 2. General population without psychiatric disorders, 3. COPD population with psychiatric disorders and 4. COPD population without psychiatric disorders) in which the association between exposure and outcomes were assessed (Figure 1).

Exposure and outcomes

We defined individuals using varenicline as the exposure group and those using NRT as the reference group. The primary outcome was incidence of any drug-treated neuropsychiatric adverse event (NPAE) including depression (drugs with ATC-codes N06A, N06CA), anxiety (N05B) and insomnia (N05C), defined as one or more new prescriptions of the specified drugs within 24 weeks after the first prescription of varenicline or NRT, considering the use of varenicline could be possibly extended to 24 weeks, which is also a suggested observed period by previous related studies.[20]

For peer review only - http://bmjopen.bmj.com/site/about/guidelines.xhtml

Covariates

The following covariates were included as possible confounders: age, gender, social economic status (SES) based on postal codes, previous psychiatric disorders and other comorbidities including heart failure, ischemic heart disease, hypertension, cancer, diabetes, osteoporosis, peptic ulcer and gastroesophageal reflux disease (GERD), rheumatic arthritis, thyroid disorder, anemia, glaucoma, gout, and allergic rhinitis. All the comorbidities were defined by at least two prescriptions of related drugs within 6 months prior to index date (see detailed ATC codes in supplementary Table S1), The ATC codes used to define the comorbidities in this study were consistent with those in previous published papers.[24-28]

Statistical methods

The continuous and categorical variables are presented as means with standard deviations (SD) and numbers with percentages, respectively. The differences of characteristics between two groups were compared using student's t-tests and chi-square tests for continuous and categorical variables, respectively. Binary logistic regression modeling was used to obtain the odds ratio (OR) and corresponding 95% confidence interval (CI) after adjustment for the potential confounders. A two-sided $p \leq 0.05$ was considered to be statistically significant for all tests. All analyses were performed using IBM SPSS statistics 25 (IBM Corporation, Armonk, NY, USA) for Windows.

Subgroup and sensitivity analyses

Considering the possible influence of age and gender on the association between NPAEs and varenicline use, we further analyzed the primary outcome in the four cohorts (general and COPD populations with or without psychiatric disease, separately) by stratifying the results by age groups and gender. To further test the robustness of the results, we performed several sensitivity analyses. First, because the possible treatment duration of varenicline is just 12 weeks, we explored the outcome occurring within 12 weeks after treatment initiation. Second, to exclude an active psychiatric status, we further selected subjects who were not prescribed any drugs for psychiatric disorders within 30 days before index date. Finally, considering the influence of policy changes about reimbursement of smoking cessation treatment on the

For peer review only - http://bmjopen.bmj.com/site/about/guidelines.xhtml

use of anti-smoking drugs,[29] we also performed a sensitivity analysis by excluding the patients whose prescription date of varenicline or NRT may be in the period of Dutch smoking policy changes (i.e. from July 1st 2011 to June 30, 2013).

Patient and public involvement

Patients were not involved in the design or conduction of the study, as this research was based on established Cohort and previously collected data. The results of this study will be distributed in scientific reports or academic conferences to benefit policy-makers, clinicians and patients.

Results

Baseline characteristics

In total, we included 9077 subjects who initiated varenicline or NRT from the general population, of which 2627 had psychiatric disorders (varenicline vs NRT: 1427 vs 1200), while 6450 did not have psychiatric disorder (varenicline vs NRT: 4480 vs 1970). For the COPD population, we included 1598 individuals, of which 649 had psychiatric disorders (varenicline vs NRT: 327 vs 322), 949 did not have psychiatric disorders (varenicline vs NRT: 608 vs 341). In both the general and COPD population, individuals treated with varenicline were younger than those treated with NRT (Table 1). Drug use for heart failure and ischemic heart disease was lower in the varenicline-treated than the NRT-treated group. In patients without psychiatric disorders, drug use for other comorbidities (e.g. diabetes and osteoporosis) was also less in individuals treated with varenicline than in those with NRT.

Primary outcome in the general and COPD population

In the general population with psychiatric disorders, the incidence of overall NPAEs within 24 weeks was lower in the varenicline group than the NRT group (75.3% vs 78.5%, OR 0.83 [0.69, 1.00], Table 2). After adjusting for potential confounders, the association did not substantially change (adjusted OR (aOR) 0.82, 95% CI [0.68, 0.99]). All the specific NPAEs were also less in the varenicline group than the NRT group, although the difference between the two comparison groups for depression and insomnia events did not reach statistical significance.

For peer review only - http://bmjopen.bmj.com/site/about/guidelines.xhtml

In the general population without psychiatric disorders, the incidence rates of NPAEs were lower than among those with psychiatric disorders. The incidence of overall NPAEs within 24-weeks in the varenicline group was lower than those in the NRT group (10.5% and 12.6%, respectively; aOR 0.85, 95% CI [0.72, 1.00]). No difference was observed between the two treatment groups for depression and anxiety, however, less insomnia was seen for varenicline than NRT (aOR was 0.63, 95% CI [0.49, 0.82]).

In the COPD population, we did not see a statistical significant difference for incidence of overall NPAEs between the varenicline and NRT groups for both the psychiatric cohort (aOR 0.97, 95% CI [0.66, 1.44], Table 3) and the non-psychiatric cohort (aOR 0.81, 95% CI [0.54, 1.20]). There were also no differences for specific NPAEs between treatment groups in these two cohorts except for anxiety, which was observed significantly less in the varenicline group compared with the NRT group (aOR 0.68, 95% CI [0.49, 0.94] for the psychiatric cohort only.

Subgroup analysis

In the general population with psychiatric disorders, the risk of overall NPAEs was even lower among varenicline than NRT users for younger patients (aOR 0.54, 95% CI [0.28, 1.04] for age $<$ 40, aOR 0.78, 95% CI [0.63, 0.98] for age 40-65, Table S2) and females (aOR 0.74, 95% CI [0.57, 0.97]). However, in the general population without psychiatric disorders, a lower risk of overall NPAE by varenicline treatment compared with NRT was seen in older patients (aOR 0.53, 95% CI [0.34, 0.83]) and male subjects (aOR 0.78, 95% CI [0.61, 0.99]). In the COPD population, in both the psychiatric and non-psychiatric cohorts, there was no difference for overall NPAEs between the varenicline and NRT groups in each age group and gender group (Table S2).

Sensitivity analysis

In the general population, considering a follow-up of 12 weeks, the risk of overall NPAEs was less for varenicline compared with NRT in both the psychiatric and non-psychiatric cohorts (aOR 0.78, 95% CI: 0.64 to 0.94; aOR 0.74, 95% CI: 0.62 to 0.89; respectively, Table S3). After limiting the study population to those who were not prescribed drugs for any psychiatric disorder or those who were not prescribed drugs for depression, anxiety and insomnia within 1 month before index date, there was no statistical

For peer review only - http://bmjopen.bmj.com/site/about/guidelines.xhtml

significant difference for overall NPAEs, irrespective of presence of psychiatric disorders in the previous year (Table S4). The result was similar when we limited our cohort to individuals whose study period was not in the period of Dutch smoking policy change; the aOR for varenicline compared with NRT was 0.86, 95% CI [0.69, 1.07] in the psychiatric cohort and 0.86, 95% CI [0.71, 1.03] in non-psychiatric cohort.

In the COPD population, there were no statistical significant differences for overall and subgroup NPAEs within follow-up of 12 weeks between the two treatments except for anxiety in the psychiatric cohort (aOR 0.64, 95% CI [0.46, 0.90], Table S5). After limiting the study population to those who were not prescribed drugs for any psychiatric disorder or those who were not prescribed drugs for depression, anxiety and insomnia within 1 month before index date, there was no statistical significant difference for overall NPAEs, irrespective of presence of psychiatric disorders (Table S6). Similar results were seen when limiting individuals to those whose study period was not in the period of Dutch smoking policy change (OR 0.97, 95% CI: 0.62 to 1.51; OR 1.00, 95% CI: 0.64 to 1.58; respectively for the psychiatric and non-psychiatric cohorts, Table S6)

Discussion

Main findings and interpretation

Within 24 weeks following initiation of varenicline treatment, we found no significantly increased risk of NPAEs in both the general and COPD population compared with those using NRT, irrespective of the presence of psychiatric disorders. These findings are consistent with the results of previous RCTs and large observational studies,[10,30,31] Considering the fact that the smoking cessation treatment may last for only 12 weeks without further treatment,[32] we also explored the NPAEs in this shorter time period and observed no increased risk in overall and specific NPAEs for varenicline compared with NRT.

In contrast to the concerns about a possible increased risk of NPAEs among varenicline users, we found a 19% and 22% relative decrease in NPAEs in varenicline users of the general population with and without psychiatric disorders, respectively, compared with NRT. Regarding the safety of varenicline for specific NPAEs, we recorded a 29% reduced risk of anxiety by varenicline (vs NRT) in the psychiatric

For peer review only - http://bmjopen.bmj.com/site/about/guidelines.xhtml

cohort, and a 37% reduced risk of insomnia in the non-psychiatric cohort. Rates of depression events were comparable between the two groups in both psychiatric and non-psychiatric cohorts among the general population. These results were consistent with the pooled results of 39 RCTs in a meta-analysis,[14] which indicated that less anxiety (hazard ratio (HR) 0.75, 95% CI: 0.61, 0.93) was also observed in the varenicline group (vs NRT), and depression episodes were also evenly distributed among two treatments (HR 0.96, 95% CI: 0.75, 1.22). Compared with our study, the difference is that in this review an increased risk of insomnia was observed in the varenicline group (HR 1.56, 95% CI: 1.36, 1.78).[14] Of note, the aforementioned review did not explore the risk of varenicline on NPAEs separately in those with and without psychiatric disorders, which may contribute to the observed differences in this review compared to our results. Of note, our result is consistent with another cohort study based data from the general practice that no increased depression were observed to be associated with varenicline (HR 0.88 [0.77-1.00]). [33]

COPD patients are considered a high-risk population with high prevalence of smoking and relatively older age, making these persons more susceptible for possible adverse drug reactions (ADEs).[17,34] Of note, in our study we did not observe an increased risk of overall NPAEs among COPD patients using varenicline in both the psychiatric and non-psychiatric cohorts. Of note, regarding the occurrence of specific NPAEs, less anxiety was seen in the varenicline group than in the NRT group in both cohorts. The safety of varenicline was not fully explored among COPD patients in previous studies. To the best of our knowledge, only two studies (one RCT and one cohort study) were previously conducted.[20,35] Similar to our results, both of these two studies did not find an increased risk of NPAEs for varenicline. Notably, in the cohort study even a reduced risk of depression was observed in varenicline users compared with users of NRT among COPD patients.[20] This may be misled by unmeasured confounders, however, after modelling the effects of possible unmeasured confounders, the author concluded that an increased risk of these adverse events was very unlikely.[20]

It is notable that there is a large heterogeneity in the definition of NPAEs across studies. In some studies, the investigators focused on moderate to serious adverse events like depression, suicide or mental disorders that require hospitalization or an emergency department visit.[20,31,36,37] While other studies

For peer review only - http://bmjopen.bmj.com/site/about/guidelines.xhtml

included all adverse symptoms (e.g. angry, nervousness) or adverse events such as traffic offences.[10,32] In this study, we used prescriptions to define neuropsychiatric outcomes for the most commonly reported NPAEs including depression, anxiety and insomnia during the study period. Despite differences in clinical definitions, the observed 24-week event rates of specific NPAEs (3%-9%) for depression, anxiety or insomnia in our general population without psychiatric disorders were similar to previous studies.[14] However, the rate of NPAEs was substantially higher in participants with psychiatric disorders than those without such illness, which was also consistent with findings from previous studies.[37-39] When we further limited our study population to those who did not experience any psychiatric disorder or not experienced any depression, anxiety and insomnia within 1 month of enrollment, we found that both the overall NPAEs and specific NPAE reduced substantially.

Although the rates of NPAEs are different between psychiatric and non-psychiatric cohorts in this study, the presence of psychiatric disorders did not influence the risk of NPAEs by varenicline compared with NRT which was also consistent with previous studies.[10,40] In a prospective longitudinal study among psychiatric patients, there was no exacerbation of psychiatric symptoms detected except gastrointestinal adverse events.[41] However, although it is not within the scope of this study, what need to be mentioned is that an increased rate of outpatient visits for schizophrenia was previously reported to be present only in patients with a pre-existing mental health disorder.[36] This may be explained by mediation through individual genetic liability.[42]

In interpreting the absence of increased risks by varenicline (vs NRT) observed in the general population, we can only speculate that the positive effect from varenicline may result from its effect of consistently reducing withdrawal-related symptoms of negative affect and raised levels of positive affect.[43] It has been reported that varenicline yields higher abstinence rates than NRT,[10,11] irrespective of smoker characteristics.[44] The successful quitting of smoking by varenicline may offer more benefits to the psychiatric status compared with NRT. Moreover, there is evidence that quitting smoking is associated with recovery in stress, anxiety and depression in smokers.[45,46] A significant and progressive improvement of anxiety and depression was also reported in an observational study, and the protective effect was observed regardless of the presence of psychiatric pathology.[40]

For peer review only - http://bmjopen.bmj.com/site/about/guidelines.xhtml

Many studies demonstrated gender differences in varenicline efficacy for smoking cessation,[47,48] some studies also found a difference in neuropsychiatric events between genders.[49] In this study, we found less risk of NPAEs by varenicline for females in the psychiatric cohort compared with NRT. This may be explained by the better therapeutic response to varenicline in women compared with men. [47] However, in the non-psychiatric cohort, we observed less risk of NPAEs in males by varenicline (vs NRT) which we cannot explain and more research needs to be done on gender disparities. Similarly, there are some indications for age group-dependent NPAEs risk by varenicline (vs NRT) in two cohorts of this study. In the psychiatric cohort, there were lower event rates in the varenicline group than the NRT group in younger age groups. Contrary, in the non-psychiatric cohort, lower event rates were seen in older users of varenicline than NRT. The age-specific effectiveness of varenicline relative to NRT patch or gum were also reported that only younger smokers achieved greater likelihood of abstinence than NRTs.[50]As such, age disparities also need to be studied more closely.

Strengths and limitations

A major strength of this study is that we evaluated the safety of varenicline in both the general and COPD population based on large real-life population data making the results representative and more applicable to daily clinical practice. Both short- (12 weeks) and long-term (24 weeks) NPAEs after treatment initiation were explored in this study. Besides the influence of current or previous psychiatric disorders, we also evaluated the influence of age and gender on the NPAEs between treatment groups. We excluded the influence of a psychiatric disorder status on the outcomes in the two comparison groups by classifying the population into two cohorts (psychiatric cohort and non-psychiatric cohort) in the stage of study design. Additionally, to test the robustness of our study results, sensitivity analyses and propensity score analysis were conducted.

Like the limitations mentioned in two previous related observational studies,[30,31] our study also has the issue of potential of unmeasured healthy user bias, although we tried our best to exclude the influence of measured baseline differences between exposure groups by adjusting for them. These unmeasured confounders could be controlled by some novel methods such as instrumental variable analysis, however, this analysis is not available in this study due to lack of a proper instrumental variable. Second, as

For peer review only - http://bmjopen.bmj.com/site/about/guidelines.xhtml

outcome events and comorbidities were defined by prescriptions of related drugs as proxies. it may have led to some misclassification. Besides, the prescription of medication may not always lead to intake of the drugs (non-adherence) and such misclassification, if random, may have caused associations to be biased towards the null value. Third, some serious behavioral changes like self-harm or suicide and minor symptoms could not be evaluated in this study due to the limitation of the prescription database, thus we only included the most frequently reported NPAEs that are commonly drug treated.

Conclusion

From this population-based real-life inception cohort study, we conclude that varenicline is unlikely associated with a substantial increased risk of NPAEs in both general and COPD patients with or without psychiatric disorders following its initiation compared with NRT. These results provide further support for the safety of varenicline to quit smoking in both the general and COPD populations.

For peer review only

For peer review only - http://bmjopen.bmj.com/site/about/guidelines.xhtml

Acknowledgement

The authors would like to thank IADB.nl pharmacies for generous contributions of providing their data for research.

Contributions

YW and EH are involved in designing the study, JHB contributed the data collection, YW analyzed the data and drafted the article. All authors contributed to the interpretation of results and revision of the manuscript. All authors read and approved the final manuscript.

Funding

This study was supported by internal funding. YW got the funding from China Scholarship Council (file no: 201506010259) for her PhD study. The funders had no role in study design, data collection and analysis, decision to publish, or preparation of the manuscript.

Competing interests

None declared.

Data sharing statement

Data are available upon reasonable request.

Ethics approval

This study is based on established database IADB.nl, data are collected in accordance with the national and European guidelines on privacy requirements for handling human data. The authors have no ethical conflicts to disclose. Ethics approval is not needed and required for this study.

Supplementary data

Table S1 to S6 are available as Supplementary data.

For peer review only - http://bmjopen.bmj.com/site/about/guidelines.xhtml

References

1. Banks E, Joshy G, Korda RJ, et al. Tobacco smoking and risk of 36 cardiovascular disease subtypes: fatal and non-fatal outcomes in a large prospective Australian study. *BMC Med.* 2019;17(1):128.
2. Gaudet MM, Carter BD, Brinton LA, et al. Pooled analysis of active cigarette smoking and invasive breast cancer risk in 14 cohort studies. *Int J Epidemiol.* 2017;46(3):881-893.
3. Tjora T, Hetland J, Aaro LE, Wold B, Wiium N, Overland S. The association between smoking and depression from adolescence to adulthood. *Addiction.* 2014;109(6):1022-1030.
4. Evins AE, Korhonen T, Kinnunen TH, Kaprio J. Prospective association between tobacco smoking and death by suicide: a competing risks hazard analysis in a large twin cohort with 35-year follow-up. *Psychol Med.* 2017;47(12):2143-2154.
5. Thun MJ, Carter BD, Feskanich D, et al. 50-Year Trends in Smoking-Related Mortality in the United States. *New Engl J Med.* 2013;368(4):351-364.
6. Collaborators GBDT. Smoking prevalence and attributable disease burden in 195 countries and territories, 1990-2015: a systematic analysis from the Global Burden of Disease Study 2015. *Lancet.* 2017;389(10082):1885-1906.
7. The L. Progress towards a tobacco-free world. *Lancet.* 2018;392(10141):1.
8. World Health Organisation. WHO report on the global tobacco epidemic 2019: offer help to quit tobacco use. Geneva: World Health Organisation, 2019: 17-21.
9. Mehrotra R, Yadav A, Sinha DN, et al. Smokeless tobacco control in 180 countries across the globe: call to action for full implementation of WHO FCTC measures. *Lancet Oncol.* 2019;20(4):e208-e217.
10. Anthenelli RM, Benowitz NL, West R, et al. Neuropsychiatric safety and efficacy of varenicline, bupropion, and nicotine patch in smokers with and without psychiatric disorders (EAGLES): a double-blind, randomised, placebo-controlled clinical trial. *Lancet.* 2016;387(10037):2507-2520.
11. Ebbert JO, Hughes JR, West RJ, et al. Effect of varenicline on smoking cessation through smoking reduction: a randomized clinical trial. *JAMA.* 2015;313(7):687-694.
12. Moore TJ, Furberg CD, Glenmullen J, Maltsberger JT, Singh S. Suicidal behavior and depression in smoking cessation treatments. *PLoS One.* 2011;6(11):e27016.
13. US Food and Drug Administration. Public health advisory: FDA requires new boxed warnings for the smoking cessation drugs Chantix and Zyban. http://wayback.archive-it.org/7993/20170112005513/http://www.fda.gov/Drugs/DrugSafety/PostmarketDrugSafetyInformationforPatientsandProviders/ucm169988.htm. Published July 1, 2009. Accessed June 7, 2019. .

For peer review only - http://bmjopen.bmj.com/site/about/guidelines.xhtml

14. Thomas KH, Martin RM, Knipe DW, Higgins JP, Gunnell D. Risk of neuropsychiatric adverse events associated with varenicline: systematic review and meta-analysis. *BMJ.* 2015;350:h1109.
15. Rothwell PM. External validity of randomised controlled trials: "to whom do the results of this trial apply?". *Lancet.* 2005;365(9453):82-93.
16. Lawrence D, Mitrou F, Zubrick SR. Smoking and mental illness: results from population surveys in Australia and the United States. *BMC Public Health.* 2009;9:285.
17. Wang Y, Bahar MA, Jansen AME, et al. Improving antibacterial prescribing safety in the management of COPD exacerbations: systematic review of observational and clinical studies on potential drug interactions associated with frequently prescribed antibacterials among COPD patients. *J Antimicrob Chemother.* 2019;74(10):2848-2864.
18. Garza D, Murphy M, Tseng LJ, Riordan HJ, Chatterjee A. A double-blind randomized placebo-controlled pilot study of neuropsychiatric adverse events in abstinent smokers treated with varenicline or placebo. *Biol Psychiatry.* 2011;69(11):1075-1082.
19. Tonstad S, Davies S, Flammer M. Psychiatric Adverse Events in Randomized, Double-Blind, Placebo-Controlled Clinical Trials of Varenicline: A Pooled Analysis (vol 33, pg 298, 2010). *Drug Safety.* 2010;33(6):522-522.
20. Kotz D, Viechtbauer W, Simpson CR, van Schayck OCP, West R, Sheikh A. Cardiovascular and neuropsychiatric risks of varenicline and bupropion in smokers with chronic obstructive pulmonary disease. *Thorax.* 2017;72(10):905-911.
21. Evins AE, Benowitz NL, West R, et al. Neuropsychiatric Safety and Efficacy of Varenicline, Bupropion, and Nicotine Patch in Smokers With Psychotic, Anxiety, and Mood Disorders in the EAGLES Trial. *J Clin Psychopharmacol.* 2019;39(2):108-116.
22. Taylor GMJ, Itani T, Thomas KH, et al. Prescribing Prevalence, Effectiveness, and Mental Health Safety of Smoking Cessation Medicines in Patients With Mental Disorders. *Nicotine Tob Res.* 2020;22(1):48-57.
23. Visser ST, Schuiling-Veninga CC, Bos JH, de Jong-van den Berg LT, Postma MJ. The population-based prescription database IADB.nl: its development, usefulness in outcomes research and challenges. *Expert Rev Pharmacoecon Outcomes Res.* 2013;13(3):285-292.
24. Kronig M, Haverkamp C, Schulte A, et al. Diabetes and beta-adrenergic blockage are risk factors for metastatic prostate cancer. *World J Surg Oncol.* 2017;15(1):50.
25. van Boven JF, van Raaij JJ, van der Galien R, et al. Impact of multiple-dose versus single-dose inhaler devices on COPD patients' persistence with long-acting beta(2)-agonists: a dispensing database analysis. *NPJ Prim Care Respir Med.* 2014;24:14069.
26. Huber CA, Szucs TD, Rapold R, Reich O. Identifying patients with chronic conditions using pharmacy data in Switzerland: an updated mapping approach to the classification of medications. *Bmc Public Health.* 2013;13.

For peer review only - http://bmjopen.bmj.com/site/about/guidelines.xhtml

27. Monte S, Macchia A, Romero M, D'Ettorre A, Giuliani R, Tognoni G. Antidepressants and cardiovascular outcomes in patients without known cardiovascular risk. *Eur J Clin Pharmacol.* 2009;65(11):1131-1138.

28. Chini F, Pezzotti P, Orzella L, Borgia P, Guasticchi G. Can we use the pharmacy data to estimate the prevalence of chronic conditions? a comparison of multiple data sources. *Bmc Public Health.* 2011;11.

29. van Boven JF, Vemer P. Higher Adherence During Reimbursement of Pharmacological Smoking Cessation Treatments. *Nicotine Tob Res.* 2016;18(1):56-63.

30. Kotz D, Viechtbauer W, Simpson C, van Schayck OC, West R, Sheikh A. Cardiovascular and neuropsychiatric risks of varenicline: a retrospective cohort study. *Lancet Respir Med.* 2015;3(10):761-768.

31. Thomas KH, Martin RM, Davies NM, Metcalfe C, Windmeijer F, Gunnell D. Smoking cessation treatment and risk of depression, suicide, and self harm in the Clinical Practice Research Datalink: prospective cohort study. *BMJ.* 2013;347:f5704.

32. Molero Y, Lichtenstein P, Zetterqvist J, Gumpert CH, Fazel S. Varenicline and risk of psychiatric conditions, suicidal behaviour, criminal offending, and transport accidents and offences: population based cohort study. *BMJ.* 2015;350:h2388.

33. Gunnell D, Irvine D, Wise L, Davies C, Martin RM. Varenicline and suicidal behaviour: a cohort study based on data from the General Practice Research Database. *BMJ.* 2009;339:b3805.

34. Mangoni AA, Jackson SH. Age-related changes in pharmacokinetics and pharmacodynamics: basic principles and practical applications. *Br J Clin Pharmacol.* 2004;57(1):6-14.

35. Tashkin DP, Rennard S, Hays JT, Ma W, Lawrence D, Lee TC. Effects of varenicline on smoking cessation in patients with mild to moderate COPD: a randomized controlled trial. *Chest.* 2011;139(3):591-599.

36. Cunningham FE, Hur K, Dong D, et al. A comparison of neuropsychiatric adverse events during early treatment with varenicline or a nicotine patch. *Addiction.* 2016;111(7):1283-1292.

37. Pasternak B, Svanstrom H, Hviid A. Use of varenicline versus bupropion and risk of psychiatric adverse events. *Addiction.* 2013;108(7):1336-1343.

38. Kanner AM, Wuu J, Faught E, et al. A past psychiatric history may be a risk factor for topiramate-related psychiatric and cognitive adverse events. *Epilepsy Behav.* 2003;4(5):548-552.

39. Croce Nanni R, Troisi A. Maternal attachment style and psychiatric history as independent predictors of mood symptoms in the immediate postpartum period. *J Affect Disord.* 2017;212:73-77.

40. Pacheco VA, Fernandez APGB, Morales AV, Crespo EL, Garcia SM, Rubio TM. Anxiety, Depression and Tobacco Abstinence. *Adicciones.* 2017;29(4):233-244.

For peer review only - http://bmjopen.bmj.com/site/about/guidelines.xhtml

41. Raich A, Ballbe M, Nieva G, et al. Safety of Varenicline for Smoking Cessation in Psychiatric and Addicts Patients. *Subst Use Misuse.* 2016;51(5):649-657.

42. Agerbo E, Sullivan PF, Vilhjalmsson BJ, et al. Polygenic Risk Score, Parental Socioeconomic Status, Family History of Psychiatric Disorders, and the Risk for Schizophrenia: A Danish Population-Based Study and Meta-analysis. *JAMA Psychiatry.* 2015;72(7):635-641.

43. Global Strategy for the Diagnosis, Management and Prevention of COPD, Global Initiative for Chronic Obstructive Pulmonary Disease (GOLD) 2019 Report. Available from: http://goldcopd.org.

44. Walker N, Gainforth H, Kiparoglou V, Robinson H, van Woerden H, West R. Factors moderating the relative effectiveness of varenicline and nicotine replacement therapy in clients using smoking cessation services. *Addiction.* 2018;113(2):313-324.

45. Zarghami M, Taghizadeh F, Sharifpour A, Alipour A. Efficacy of Smoking Cessation on Stress, Anxiety, and Depression in Smokers with Chronic Obstructive Pulmonary Disease: A Randomized Controlled Clinical Trial. *Addict Health.* 2018;10(3):137-147.

46. Taylor G, McNeill A, Girling A, Farley A, Lindson-Hawley N, Aveyard P. Change in mental health after smoking cessation: systematic review and meta-analysis. *BMJ.* 2014;348:g1151.

47. McKee SA, Smith PH, Kaufman M, Mazure CM, Weinberger AH. Sex Differences in Varenicline Efficacy for Smoking Cessation: A Meta-Analysis. *Nicotine Tob Res.* 2016;18(5):1002-1011.

48. Smith PH, Kasza KA, Hyland A, et al. Gender differences in medication use and cigarette smoking cessation: results from the International Tobacco Control Four Country Survey. *Nicotine Tob Res.* 2015;17(4):463-472.

49. The Lancet P. Sex and gender in psychiatry. *Lancet Psychiatry.* 2016;3(11):999.

50. Chang PY, Shiu MN, Yuan YT, Chang HC, Su PY, Lan TH. Comparative Effectiveness of Varenicline and Nicotine Replacement Therapy for Smoking Cessation in Older and Younger Smokers: A Prospective Cohort in Taiwan. *Nicotine Tob Res.* 2019;21(2):149-155.

For peer review only

For peer review only - http://bmjopen.bmj.com/site/about/guidelines.xhtml

Tables and Figure

Table 1. Baseline characteristics of general and COPD population with and without psychiatric disorders by treatment groups.

Table 2. Incidence of neuropsychiatric adverse events (NPAEs) and association with varenicline compared with NRT in general populations with and without psychiatric disorders with follow up of 24 weeks.

Table 3. Incidence of neuropsychiatric adverse events (NPAEs) and association with varenicline compared with NRT in COPD population with and without psychiatric disorders within follow up of 24 weeks.

Figure 1. Flow chart of population selection. NRT: nicotine replacement therapies; ATC: anatomical therapeutic chemical.

For peer review only

For peer review only - http://bmjopen.bmj.com/site/about/guidelines.xhtml

1 **Table 1**. Baseline characteristics of general and COPD population with and without psychiatric disorders by treatment groups.

**Characteristics**	**General population (n = 9077)**				**COPD population (1598)**			
	Psychiatric cohort (n = 2627)		Non-psychiatric cohort (n = 6450)		Psychiatric cohort (n = 649)		Non-psychiatric cohort (n = 949)	
	Varenicline (N=1427)	NRT (N=1200)	Varenicline (N=4480)	NRT (N= 1970)	Varenicline (N=327)	NRT (N=322)	Varenicline (N=608)	NRT (N= 341)
**Age (years)**								
**Mean (SD)**	54.5 (9.6)*	55.8 (11.8)	52.1 (9.7)*	53.4 (12.5)	59.2 (8.6)*	62.4 (10.2)	58.9 (8.9)*	62.7 (9.8)
**Age range**	32 - 87	25 - 92	32 - 102	22 - 90	41 - 87	41 - 92	40 - 102	42 -89
**Gender (n, %)**								
**Men**	541 (37.9)*	523 (43.6)	2406 (53.7)	1100 (55.8)	111 (33.9)	126 (39.1)	310 (51.0)	184 (54.0)
**Female**	886 (62.1)	677 (56.4)	2074 (46.3)	870 (44.2)	216 (66.1)	196 (60.9)	298 (49.0)	157 (46.0)
**Year of index date (n, %)**								
**1994-2010**	519 (36.4)*	492 (41.0)	1497 (33.4)*	895 (45.4)	135 (41.3)	135 (41.9)	211 (34.7)	124 (36.4)
**2011-2017**	908 (63.6)	708 (59.0)	2983 (66.6)	1075 (54.6)	192 (58.7)	187 (58.1)	397 (65.3)	217 (63.6)
**Social economic status (n, %)**								
**Low**	714 (50.0)*	655 (54.6)	2117 (47.3)*	1080 (54.8)	170 (52.0)	172 (53.4)	287 (47.2)	175 (51.3)
**High**	713 (50.0)	545 (45.4)	2363 (52.7)	890 (45.2)	157 (48.0)	150 (46.6)	321 (52.8)	166 (48.7)
**Comorbidities (n, %)**								

For peer review only - http://bmjopen.bmj.com/site/about/guidelines.xhtml

**Heart failure**	41 (2.9)*	82 (6.8)	47 (1.0)*	53 (2.7)	23 (7.0)*	42 (13.0)	20 (3.3)*	27 (7.9)
**Ischemic heart disease**	25 (1.8)*	39 (3.3)	36 (0.8)*	31 (1.6)	9 (2.8)*	20 (6.2)	9 (1.5)*	12 (3.5)
**Hypertension**	508 (35.6)	465 (38.8)	1039 (23.2)*	547 (27.8)	146 (44.6)	167 (51.9)	234 (38.5)	145 (42.5)
**Cancers**	9 (0.6)*	0 (0.0)	9 (0.2)	9 (0.5)	0 (0.0)	0 (0.0)	0 (0.0)	2 (0.6)
**Diabetes mellitus**	162 (11.4)	129 (10.8)	260 (5.8)*	146 (7.4)	53 (16.2)	40 (12.4)	47 (7.7)*	43 (12.6)
**Osteoporosis**	39 (2.7)	32 (2.7)	25 (0.6)*	26 (1.3)	17 (5.2)	20 (6.2)	6 (1.0)*	14 (4.1)
**Peptic ulcer and GERD**	436 (30.6)	407 (33.9)	474 (10.6)*	253 (12.8)	134 (41.0)	142 (44.1)	123 (20.2)	82 (24.0)
**Rheumatic arthritis**	170 (11.9)	172 (14.3)	204 (4.6)*	123 (6.2)	45 (13.8)	41 (12.7)	40(6.6)	21 (6.2)
**Thyroid disorder**	55 (3.9)	40 (3.3)	96 (2.1)	49 (2.5)	18 (5.5)	13 (4.0)	18 (3.0)	5 (1.5)
**Anemia**	49 (3.4)	52 (4.3)	49 (1.1)*	38 (1.9)	11 (3.4)	20 (6.2)	4 (0.7)*	12 (3.5)
**Glaucoma**	13 (0.9)	12 (1.0)	17 (0.4)*	23 (1.2)	4 (1.2)	4 (1.2)	3 (0.5)*	7 (2.1)
**Gout**	4 (0.3)	9 (0.8)	16 (0.4)*	24 (1.2)	1 (0.3)	3 (0.9)	3 (0.5)*	10 (2.9)
**Allergic rhinitis**	76 (5.3)	47 (3.9)	104 (2.3)	34 (1.7)	28 (8.6)	24 (7.5)	33 (5.4)	15 (4.4)

*$p<0.05$; GERD: Gastroesophageal Reflux Disease. NRT: Nicotine replacement therapy.

For peer review only

For peer review only - http://bmjopen.bmj.com/site/about/guidelines.xhtml

4 **Table 2**. Incidence of neuropsychiatric adverse events (NPAEs) and association with varenicline compared with NRT in general population with and without
5 psychiatric disorders with follow up of 24 weeks.

NPAEs	Psychiatric cohort Varenicline (n=1427) vs NRT (n=1200)			Non-psychiatric cohort Varenicline (n=4480) vs NRT (n=1970)		
	Events (n, %)[#]	Crude OR (95% CI)	Adjusted OR[*] (95% CI)	Events (n, %)[#]	Crude OR (95% CI)	Adjusted OR[**] (95% CI)
**Overall**	1074 (75.3): 942 (78.5)	0.83 [0.69, 1.00]	0.82 [0.68, 0.99]	469 (10.5): 248 (12.6)	0.81 [0.69, 0.96]	0.85 [0.72, 1.00]
Depression	629 (44.1): 548 (45.7)	0.94 [0.80, 1.09]	0.90 [0.77, 1.05]	148 (3.3): 57 (2.9)	1.15 [0.84, 1.56]	1.15 [0.84, 1.57]
Anxiety	456 (32.0): 472 (39.3)	0.72 [0.62, 0.85]	0.71 [0.61, 0.84]	215 (4.8): 110 (5..6)	0.85 [0.67, 1.08]	0.90 [0.71, 1.15]
Insomnia	372 (26.1): 352 (29.3)	0.85 [0.72, 1.01]	0.89 [0.75, 1.06]	147 (3.3): 105 (5.3)	0.60 [0.47, 0.78]	0.63 [0.49, 0.82]

6 NPAEs: neuropsychiatric adverse events; NRT: nicotine replacement therapy; OR: odds ratio; CI: confidence interval; [*]Adjusted for age, gender, socioeconomic
7 status, heart failure, ischemic heart disease and cancer;. [**]Adjusted for age, socioeconomic status, heart failure, ischemic heart disease, hypertension, diabetes,
8 osteoporosis, peptic ulcer and GERD, Rheumatic arthritis, anemia, glaucoma and gout; [#] Varenicline: NRT;

9

For peer review only - http://bmjopen.bmj.com/site/about/guidelines.xhtml

Table 3. Incidence of neuropsychiatric adverse events (NPAEs) and association with varenicline compared with NRT in COPD population with and without psychiatric disorders with follow up of 24 weeks.

NPAEs	**Psychiatric cohort** Varenicline (n=327) vs NRT (n=322)			**Non-psychiatric cohort** Varenicline (n=608) vs NRT (n=341)		
	Events (n, %)[#]	Crude OR (95% CI)	Adjusted OR* (95% CI)	Events (n, %)[#]	Crude OR (95% CI)	Adjusted OR** (95% CI)
**Overall**	258 (78.9): 260 (80.7)	0.89 [0.61, 1.31]	0.97 [0.66, 1.44]	76 (12.5): 53 (15.5)	0.78 [0.53, 1.13]	0.81 [0.54,1.20]
Depression	146 (44.6): 145 (45.0)	0.99 [0.72, 1.34]	0.90 [0.65, 1.24]	22 (3.6): 10 (2.9)	1.24 [0.58, 2.66]	1.37 [0.62, 3.03]
Anxiety	110 (33.6): 136 (42.2)	0.69 [0.50, 0.95]	0.68 [0.49, 0.94]	38 (6.3): 25 (7.3)	0.84 [0.50, 1.42]	0.86 [0.50, 1.49]
Insomnia	102 (31.2): 124 (38.5)	0.72 [0.52, 1.00]	0.84 [0.60, 1.18]	25 (4.1): 21 (6.2)	0.65 [0.36, 1.19]	0.63 [0.34, 1.17]

NPAEs: neuropsychiatric adverse events; NRT: nicotine replacement therapy; OR: odds ratio; CI: confidence interval; *Adjusted for age, heart failure, ischemic heart disease; **Adjusted for age, heart failure, ischemic heart disease, diabetes, osteoporosis, anemia, glaucoma and gout; # varenicline:NRT.

For peer review only

For peer review only - http://bmjopen.bmj.com/site/about/guidelines.xhtml

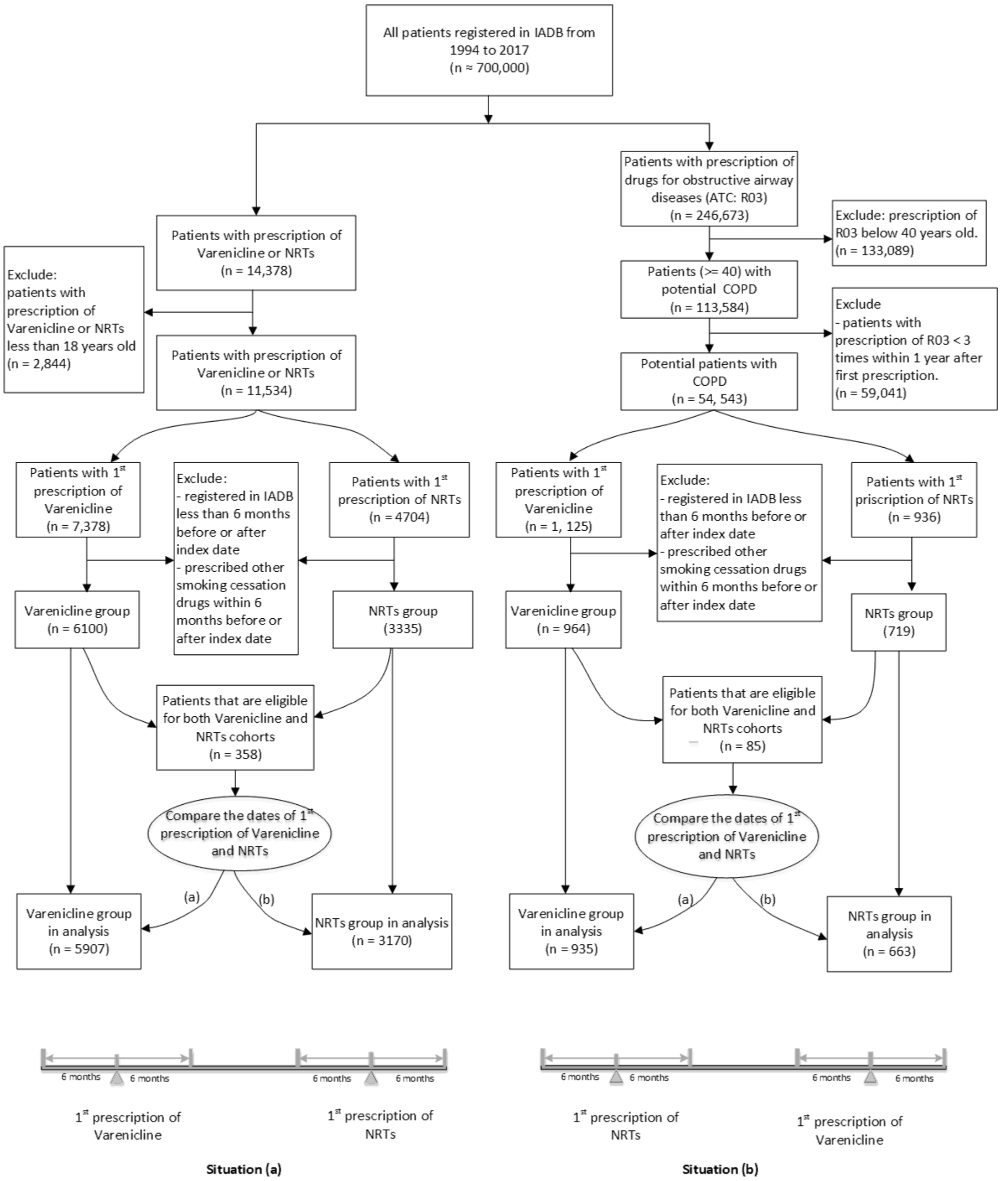

Figure 1. Flow chart of population selection. NRT: nicotine replacement therapies; ATC: anatomical therapeutic chemical.

285x338mm (106 x 106 DPI)

Supplementary Materials

Table S1. List of ATC codes used for identification of related diseases and outcome events.

Variables	ATC codes
COPD	R03
General psychiatric disorders	N02, N03, N04, N05, N06
Depression	N06A, N06CA,
Anxiety	N05B
Insomnia	N05C
Heart failure	C01AA, C03C
Ischemic heart disease	C01DA
Hypertension	C02, C03 (except C03C), C07, C08, C09
Cancers	L01
Diabetes mellitus	A10
Osteoporosis	M05B
Peptic ulcer and GERD	A02B
Rheumatic arthritis	M01, M02
Thyroid disorders	H03
Anemia	B03
Glaucoma	S01E
Gout	M04
Allergic rhinitis	R01AD

ATC: anatomical therapeutic chemical; COPD: chronic obstructive pulmonary disease; GERD: gastroesophageal reflux disease;

For peer review only

Table S2. Subgroup analysis: incidence of neuropsychiatric adverse events (NPAEs) and association with varenicline compared with NRT within follow up of 24 weeks stratified by age and gender.

Subgroups	General populations (N=9077)				COPD populations (N=1958)			
	Psychiatric cohort Varenicline(n=1427) vs NRT(1200)		Non-psychiatric cohort Varenicline(n=4480) vs NRT(n=1970)		Psychiatric cohort Varenicline(n=327) vs NRT(322)		Non-psychiatric cohort Varenicline(n=608) vs NRT(n=341)	
	Crude OR(95% CI)	aOR (95% CI)	Crude OR (95% CI)	aOR (95% CI)	Crude OR (95% CI)	aOR (95% CI)	Crude OR (95% CI)	aOR (95% CI)
Age **								
Age < 40	0.58 [0.32, 1.05]	0.54 [0.28, 1.04]	1.01 [0.61, 1.66]	0.93 [0.54, 1.64]	---	---	---	---
40-65	0.81 [0.65, 1.01]	0.78 [0.63, 0.98]	0.86 [0.71, 1.04]	0.87 [0.72, 1.07]	0.88 [0.56, 1.38]	0.87 [0.54, 1.41]	0.91 [0.57, 1.46]	0.93 [0.57, 1.53]
>65	1.02 [0.66, 1.59]	1.13 [0.70, 1.83]	0.50 [0.33, 0.78]	0.53 [0.34, 0.83]	1.08 [0.50, 2.35]	1.02 [0.43, 2.40]	0.51 [0.25, 1.05]	0.51 [0.24, 1.11]
Gender (n, %)**								
Men	0.87 [0.67, 1.13]	0.89 [0.68, 1.16]	0.71 [0.56, 0.91]	0.78 [0.61, 0.99]	0.95 [0.54, 1.69]	1.24 [0.66, 2.33]	0.81 [0.46, 1.43]	1.00 [0.53, 1.88]
Female	0.73 [0.56, 0.95]	0.74 [0.57, 0.97]	0.89 [0.71, 1.11]	0.91 [0.72, 1.14]	0.78 [0.46, 1.33]	0.77 [0.44, 1.35]	0.73 [0.44, 1.22]	0.76 [0.44, 1.31]

NPAEs: neuropsychiatric adverse events; NRTs: nicotine replacement therapy; OR: odds ratio; CI: confidence interval; aOR: adjusted odds ratio;*adjusted gender, social economic status and comorbidities; **adjusted age, social economic status, comorbidities.

For peer review only

Table S3. Sensitivity analysis: incidence of neuropsychiatric adverse events (NPAEs) and association with varenicline compared with NRT in general population with and without psychiatric disorders within follow up of 12 weeks.

**NPAEs**	**Psychiatric cohort (N=2627)** Varenicline (n=1427) vs NRTs (n=1200)			**Non-psychiatric cohort (N=6450)** Varenicline (n=4480) vs NRTs (n=1970)		
	Events (n, %)*	Crude OR (95% CI)	Adjusted OR# (95% CI)	Events (n, %)*	Crude OR (95% CI)	Adjusted OR# (95% CI)
**Follow-up of 12 weeks**						
Overall	1062 (74.4): 948 (79.0)	0.77 [0.64, 0.93]	0.78 [0.64, 0.94]	363 (8.1): 223 (11.3)	0.69 [0.58,0.82]	0.74 [0.62, 0.89]
Depression	560 (39.2): 491 (40.9)	0.93[0.80, 1.09]	0.89 [0.76, 1.05]	86 (1.9): 30 (1.5)	1.27 [0.83, 1.92]	1.29 [0.84, 1.97]
Anxiety	367 (25.7): 423 (35.3)	0.64 [0.54, 0.75]	0.63 [0.53, 0.74]	119 (2.7): 61 (3.1)	0.85 (0.62, 1.17)	0.90 [0.65, 1.24]
Insomnia	321 (22.5): 311 (25.9)	0.83 [0.69, 0.99]	0.88 [0.73, 1.05]	82 (1.8): 61 (3.1)	0.58 [0.42, 0.82]	0.62 [0.44, 0.88]

NPAE: neuropsychiatric adverse events; OR: odds ratio; CI: confidence interval; NRTs: nicotine replacement therapy; # adjusted for age, gender, social economic status and related comorbidities. *Varenicline:NRT.

For peer review only

Table S4. Sensitivity analysis: incidence of neuropsychiatric adverse events (NPAEs) and association with compared with NRT in general population within follow up of 24 weeks.

Limitations	General populations (N=9077)					
	Psychiatric cohort varenicline(n=1427) vs NRTs (1200)			Non-psychiatric cohort varenicline(n=4480) vs NRTs(1970)		
	Events (n, %)[#]	OR (95% CI)	aOR (95% CI)[*]	Events (n, %)[#]	OR (95% CI)	aOR (95% CI)[*]
**Exclude participants prescribed drugs for any NSP within 1 month before index date**						
Overall	254 (57.6): 157 (60.4)	0.89 [0.65, 1.22]	0.87 [0.63, 1.21]	418 (9.6): 192 (10.4)	0.91 [0.76, 1.10]	0.93 [0.78, 1.12]
Depression	158 (35.8): 82 (31.5)	1.21 [0.88, 1.68]	1.18 [0.84, 1.67]	139 (3.2): 50 (2.7)	1.18 [0.85, 1.64]	1.16 [0.84, 1.62]
Anxiety	78 (17.7): 62 (23.8)	0.69 [0.47, 0.10]	0.68 [0.46, 1.01]	197 (4.5): 85 (4.6)	0.98 [0.76, 1.27]	1.02 [0.78, 1.33]
Insomina	75 (17.0): 48 (18.5)	0.94 [0.74, 1.20]	0.94 [0.62, 1.43]	131 (3.0): 91 (4.9)	0.60 [0.46, 0.79]	0.61 [0.46, 0.80]
**Exclude participants prescribed drugs for depression, anxiety and insomnia within 1 month before index date**						
Overall	314 (48.9): 201 (46.1)	1.12 [0.88, 1.43]	1.05 [0.82, 1.36]	424 (9.6): 203 (10.6)	0.90 [0.75, 1.07]	0.92 [0.77, 1.11]
Depression	191 (29.8): 104 (23.9)	1.35 [1.02, 1.79]	1.25 [0.93, 1.66]	143 (3.3): 52 (2.7)	1.20 [0.87, 1.66]	1.19 [0.86, 1.65]
Anxiety	99 (15.4): 81 (18.6)	0.80 [0.58, 1.10]	0.75 [0.54, 1.05]	199 (4.5): 92 (4.8)	0.94 [0.73, 1.21]	0.98 [0.75, 1.26]
Insomnia	92 (14.3): 63 (14.4)	0.99 [0.70, 1.40]	1.03 [0.72, 1.47]	132 (3.0): 94 (4.9)	0.60 [0.46, 0.78]	0.61 [0.47, 0.81]
**Exclude participants whose study period includes policies changes**						
Overall	797 (76.0): 733 (77.7)	0.91 [0.74, 1.12]	0.86 [0.69, 1.07]	352 (11.1): 205 (12.9)	0.85 [0.70, 1.02]	0.86 [0.71, 1.03]
Depression	468 (44.7): 411 (43.6)	1.04 [0.88, 1.25]	0.98 [0.82, 1.18]	115 (3.6): 45 (2.8)	1.30 [0.91, 1.84]	1.28 [0.90, 1.82]
Anxiety	343 (32.7): 372 (39.4)	0.75 [0.62, 0.90]	0.72 [0.60, 0.87]	169 (5.3): 95 (6.0)	0.89 [0.69, 1.15]	0.91 [0.70, 1.19]
Insomnia	273 (26.0): 280 (29.7)	0.83 [0.69, 1.02]	0.87 [0.71, 1.07]	109 (3.4): 83 (5.2)	0.65 [0.48, 0.87]	0.66 [0.49, 0.88]

NPAEs: neuropsychiatric adverse events; NRTs: nicotine replacement therapy; OR: odds ratio; aOR: adjusted odds ratio; CI: confidence interval; [*]adjusted age, gender, social economic status and comorbidities. We set the indexdate was within period between July 1st, 2011 and June 30, 2013; Policy change: In the Netherlands, pharmacologic

For peer review only - http://bmjopen.bmj.com/site/about/guidelines.xhtml

Smoking Cessation Treatments (pSCTs) were reimbursed in 2011. In 2012 the reimbursement was discontinued. As of 2013, pSCTs were again reimbursed, provided they are accompanied by behavioural counselling. #Varenicline: NRT.

For peer review only

Table S5. Sensitivity analysis: incidence of neuropsychiatric adverse events (NPAEs) and association with varenicline compared with NRT in COPD population with and without psychiatric disorders within follow up of 12 weeks.

Outcomes	**Psychiatric cohort (N=649)** Varenicline (n=327) vs NRTs (n=322)			**Non-psychiatric cohort (N=949)** Varenicline (n=608) vs NRTs (n=341)		
	Events (n, %)[#]	Crude OR (95% CI)	Adjusted OR[*] (95% CI)	Events (n, %)[#]	Crude OR (95% CI)	Adjusted OR[*] (95% CI)
**Follow-up of 12 weeks**						
Overall	260 (79.5): 267 (82.9)	0.80 [0.54, 1.19]	0.89 [0.59, 1.36]	63 (10.4): 45 (13.2)	0.76 [0.51, 1.14]	0.84 [0.54, 1.29]
Depression	134 (41.0): 127 (39.4)	1.07 [0.78, 1.46]	0.94 [0.67, 1.32]	13 (2.1): 5 (1.5)	1.47 [0.52, 4.15]	1.95 [0.65, 5.91]
Anxiety	95 (29.1): 123 (38.2)	0.66 [0.48, 0.92]	0.64 [0.45, 0.90]	16 (2.6): 13 (3.8)	0.68 [0.32, 1.44]	0.68 [0.31, 1.52]
Insomnia	88 (26.9): 114 (35.4)	0.67 [0.48, 0.94]	0.72 [0.51, 1.03]	20 (3.3): 11 (3.2)	1.02 [0.48, 2.16]	1.12 [0.51, 2.46]

NPAE: neuropsychiatric adverse events; OR: odds ratio; CI: confidence interval; NRTs: nicotine replacement therapy; [*]adjusted for age, gender, social economic status and related comorbidities. [#]Varenicline:NRT.

For peer review only

Table S6. Sensitivity analysis: incidence of neuropsychiatric adverse events (NPAEs) and association with compared with NRT in COPD population within follow up of 24 weeks.

Limitations	COPD populations					
	Psychiatric cohort (N=649) varenicline(n=327) vs NRTs(322)			Non-psychiatric cohort (N=949) varenicline(608) vs NRTs(341)		
	Events (n, %)#	OR (95% CI)	aOR (95% CI)*	Events (n, %)#	OR (95% CI)	aOR (95% CI)*
**Exclude participants prescribed drugs for any NSP within 1 month before index date**						
Overall	54 (60.0): 39 (61.9)	0.92 [0.48, 1.79]	0.85 [0.41, 1.77]	72 (12.1): 40 (12.7)	0.95 [0.63, 1.43]	0.92 [0.60, 1.43]
Depression	29 (32.2): 19 (30.2)	1.10 [0.55, 2.21]	0.88. [0.39, 1.97]	20 (3.4): 10 (3.2)	1.06 [0.49, 2.29]	1.12 [0.50, 2.52]
Anxiety	22 (24.4): 20 (31.7)	0.70 [0.34, 1.42]	0.87 [0.40, 1.90]	38 (6.4): 19 (6.0)	1.06 [0.60, 1.87]	1.10 [0.60, 2.01]
Insomina	15 (16.7): 16 (25.4)	0.59 [0.27, 1.30]	0.60 [0.24, 1.49]	25 (4.2): 16 (5.1)	0.82 [0.43, 1.56]	0.71 [0.36, 1.37]
**Exclude participants prescribed drugs for depression, anxiety and insomnia within 1 month before index date**						
Overall	68 (51.1): 48 (47.5)	1.16 [0.69, 1.94]	1.29 [0.73, 2.26]	73 (12.1): 43 (13.0)	0.92 [0.62, 1.38]	0.93 [0.61, 1.42]
Depression	35 (26.3): 23 (22.8)	1.21 [0.66, 2.22]	1.08 [0.56, 2.08]	21 (3.5): 10 (3.0)	1.16 [0.54, 2.49]	1.33 [0.60, 2.99]
Anxiety	27 (20.3): 23 (22.8)	0.86 [0.46, 1.62]	0.97 [0.49, 1.91]	38 (6.3): 21 (6.4)	0.99 [0.57, 1.72]	1.00 [0.56, 1.79]
Insomnia	21 (15.8): 20 (19.8)	0.76 [0.39, 1.49]	0.87 [0.42, 1.82]	25 (4.2): 17 (5.2)	0.80 [0.43, 1.50]	0.72 [0.37, 1.39]
**Exclude participants whose study period includes policies changes**						
Overall	203 (78.1): 211 (80.2)	0.88 [0.58, 1.34]	0.97 [0.62, 1.51]	63 (14.2): 41 (15.1)	0.93 [0.61, 1.43]	1.00 [0.64, 1.58]
Depression	114 (43.8): 110 (41.8)	1.09 [0.77, 1.54]	0.96 [0.66, 1.40]	18 (4.1): 7 (2.6)	1.60 [0.66, 3.89]	1.73 [0.68, 4.42]
Anxiety	89 (34.2): 109 (41.4)	0.74 [0.52, 1.05]	0.72 [0.49, 1.05]	35 (7.9): 20 (7.4)	1.08 [0.61, 1.91]	1.21 [0.66, 2.24]
Insomnia	81 (31.2): 106 (40.3)	0.67 [0.47, 0.96]	0.75 [0.51, 1.10]	19 (4.3): 15 (5.5)	0.77 [0.38, 0.53]	0.71 [0.34, 1.45]

NPAEs: neuropsychiatric adverse events; NRTs: nicotine replacement therapy; OR: odds ratio; *aOR: adjusted odds ratio; *adjusted age, gender, social economic status and comorbidities. # Varenicline: NRT. We set the indexdate was within period between July 1st, 2011 and June 30, 2013; Policy change: In the Netherlands, pharmacologic

For peer review only

Smoking Cessation Treatments (pSCTs) were reimbursed in 2011. In 2012 the reimbursement was discontinued. As of 2013, pSCTs were again reimbursed, provided they are accompanied by behavioural counselling.

For peer review only

STROBE Statement—Checklist of items that should be included in reports of cohort studies

	Item No	Recommendation	Page No
**Title and abstract**	1	(*a*) Indicate the study's design with a commonly used term in the title or the abstract	1
		(*b*) Provide in the abstract an informative and balanced summary of what was done and what was found	2
**Introduction**			
Background/rationale	2	Explain the scientific background and rationale for the investigation being reported	4
Objectives	3	State specific objectives, including any prespecified hypotheses	5
**Methods**			
Study design	4	Present key elements of study design early in the paper	5
Setting	5	Describe the setting, locations, and relevant dates, including periods of recruitment, exposure, follow-up, and data collection	5,6,7
Participants	6	(*a*) Give the eligibility criteria, and the sources and methods of selection of participants. Describe methods of follow-up	5,6
		(*b*) For matched studies, give matching criteria and number of exposed and unexposed	N/A
Variables	7	Clearly define all outcomes, exposures, predictors, potential confounders, and effect modifiers. Give diagnostic criteria, if applicable	6, 7
Data sources/ measurement	8*	For each variable of interest, give sources of data and details of methods of assessment (measurement). Describe comparability of assessment methods if there is more than one group	5, 6, 7
Bias	9	Describe any efforts to address potential sources of bias	6, 7
Study size	10	Explain how the study size was arrived at	N/A
Quantitative variables	11	Explain how quantitative variables were handled in the analyses. If applicable, describe which groupings were chosen and why	7
Statistical methods	12	(*a*) Describe all statistical methods, including those used to control for confounding	6,7,8
		(*b*) Describe any methods used to examine subgroups and interactions	6,7
		(*c*) Explain how missing data were addressed	N/A
		(*d*) If applicable, explain how loss to follow-up was addressed	N/A
		(*e*) Describe any sensitivity analyses	7
**Results**			
Participants	13*	(a) Report numbers of individuals at each stage of study—eg numbers potentially eligible, examined for eligibility, confirmed eligible, included in the study, completing follow-up, and analysed	8
		(b) Give reasons for non-participation at each stage	8
		(c) Consider use of a flow diagram	20
Descriptive data	14*	(a) Give characteristics of study participants (eg demographic, clinical, social) and information on exposures and potential confounders	8
		(b) Indicate number of participants with missing data for each variable of interest	N/A
		(c) Summarise follow-up time (eg, average and total amount)	8,9
Outcome data	15*	Report numbers of outcome events or summary measures over time	8, 9,10, 20
**Discussion**			

Key results	18	Summarise key results with reference to study objectives	10,11,12
Limitations	19	Discuss limitations of the study, taking into account sources of potential bias or imprecision. Discuss both direction and magnitude of any potential bias	13, 14
Interpretation	20	Give a cautious overall interpretation of results considering objectives, limitations, multiplicity of analyses, results from similar studies, and other relevant evidence	10,11,12, 13
Generalisability	21	Discuss the generalisability (external validity) of the study results	13
**Other information**			
Funding	22	Give the source of funding and the role of the funders for the present study and, if applicable, for the original study on which the present article is based	15

*Give information separately for exposed and unexposed groups.

Note: An Explanation and Elaboration article discusses each checklist item and gives methodological background and published examples of transparent reporting. The STROBE checklist is best used in conjunction with this article (freely available on the Web sites of PLoS Medicine at http://www.plosmedicine.org/, Annals of Internal Medicine at http://www.annals.org/, and Epidemiology at http://www.epidem.com/). Information on the STROBE Initiative is available at http://www.strobe-statement.org.

For peer review only

BMJ Open

Neuropsychiatric safety of varenicline in the general and COPD population with and without psychiatric disorders: a retrospective cohort study in a real-world setting

Journal:	*BMJ Open*
Manuscript ID	bmjopen-2020-042417.R1
Article Type:	Original research
Date Submitted by the Author:	13-Jan-2021
Complete List of Authors:	Wang, Yuanyuan; University of Groningen, Groningen Research Institute of Pharmacy, Department of PharmacoTherapy, -Epidemiology and -Economics<br>Bos, Jens; University of Groningen Groningen Research Institute of Pharmacy, Department of PharmacoTherapy, -Epidemiology & -Economics<br>Schuiling-Veninga, Catharina; University of Groningen Groningen Research Institute of Pharmacy, Department of PharmacoTherapy, -Epidemiology & -Economics<br>Boezen, H. Marike; University of Groningen, University Medical Center Groningen, Department of Epidemiology; University of Groningen, University medical center Groningen, Groningen Research Institute for Asthma and COPD (GRIAC)<br>van Boven, Job F. M.; University of Groningen, University Medical Center Groningen, Groningen Research Institute for Athma and COPD (GRIAC) ; University of Groningen, University Medical Center Groningen, Department of Clinical Pharmacy & Pharmacology<br>Wilffert, B; Rijksuniversiteit Groningen,<br>Hak, Eelko; University of Groningen Groningen Research Institute of Pharmacy, Department of PharmacoTherapy, -Epidemiology & -Economics; University of groningen, University Medical Center Groningen, Deparment of Epidemiology
**Primary Subject Heading**:	Smoking and tobacco
Secondary Subject Heading:	Addiction
Keywords:	Depression & mood disorders < PSYCHIATRY, Anxiety disorders < PSYCHIATRY, EPIDEMIOLOGY

SCHOLARONE™
Manuscripts

I, the Submitting Author has the right to grant and does grant on behalf of all authors of the Work (as defined in the below author licence), an exclusive licence and/or a non-exclusive licence for contributions from authors who are: i) UK Crown employees; ii) where BMJ has agreed a CC-BY licence shall apply, and/or iii) in accordance with the terms applicable for US Federal Government officers or employees acting as part of their official duties; on a worldwide, perpetual, irrevocable, royalty-free basis to BMJ Publishing Group Ltd ("BMJ") its licensees and where the relevant Journal is co-owned by BMJ to the co-owners of the Journal, to publish the Work in this journal and any other BMJ products and to exploit all rights, as set out in our licence.

The Submitting Author accepts and understands that any supply made under these terms is made by BMJ to the Submitting Author unless you are acting as an employee on behalf of your employer or a postgraduate student of an affiliated institution which is paying any applicable article publishing charge ("APC") for Open Access articles. Where the Submitting Author wishes to make the Work available on an Open Access basis (and intends to pay the relevant APC), the terms of reuse of such Open Access shall be governed by a Creative Commons licence – details of these licences and which Creative Commons licence will apply to this Work are set out in our licence referred to above.

Other than as permitted in any relevant BMJ Author's Self Archiving Policies, I confirm this Work has not been accepted for publication elsewhere, is not being considered for publication elsewhere and does not duplicate material already published. I confirm all authors consent to publication of this Work and authorise the granting of this licence.

For peer review only

Neuropsychiatric safety of varenicline in the general and COPD population with and without psychiatric disorders: a retrospective cohort study in a real-world setting

Yuanyuan Wang[1], Jens H. Bos[1], Catharina C.M. Schuiling-Veninga[1], H. Marike Boezen[2,3], Job F.M. van Boven[3,4], Bob Wilffert[1,4], Eelko Hak[1,2]

[1]University of Groningen, Groningen Research Institute of Pharmacy, Department of PharmacoTherapy, -Epidemiology & -Economics, Groningen, The Netherlands

[2]University of Groningen, University Medical Center Groningen, Department of Epidemiology, Groningen, The Netherlands.

[3]University of Groningen, University Medical Center Groningen, Groningen Research Institute for Asthma and COPD (GRIAC), Groningen, Netherlands.

[4]University of Groningen, University Medical Center Groningen, Department of Clinical Pharmacy & Pharmacology, Groningen, The Netherlands.

*Corresponding author: Dr. Yuanyuan Wang,

Department of PharmacoTherapy, -Epidemiology & -Economics,

Groningen Research Institute of Pharmacy, University of Groningen.

P.O. BOX.196, 9700 AD Groningen, The Netherlands.

Tel. +31 (0) 50-363-9163; Fax: +31 (0) 50-363-2772.

Email: yuanyuan.wang@rug.nl; yuanyuanwang.research@gmail.com;

Keywords: smoking cessation; varenicline; nicotine replacement therapy; adverse drug events;

Text word count: 4025

Abstract word: 300

For peer review only

For peer review only - http://bmjopen.bmj.com/site/about/guidelines.xhtml

Abstract

Objectives: To evaluate the real-world association between varenicline and neuropsychiatric adverse events (NPAEs) in general and COPD population with and without psychiatric disorders compared with nicotine replacement therapy (NRT) to strengthen the knowledge of varenicline safety.

Design: A retrospective cohort study.

Setting: Prescription database IADB.nl, Netherlands.

Participants: New users of varenicline or NRT among general (≥18 years) and COPD (≥ 40 years) population. Psychiatric sub-cohort was defined as people prescribed psychotropic medications (≥ 2) within 6 months before the index date.

Outcome measures: The incidence of NPAEs including depression, anxiety and insomnia, defined by new or naive prescriptions of related medications in IADB.nl within 24 weeks after first treatment initiation of varenicline or NRT.

Results: For the general population in psychiatric cohort, the incidence of total NPAEs in varenicline (4480) and NRT (1970) groups was 10.5% and 12.6%, respectively (adjusted OR [aOR] 0.85, 95% CI 0.72 to 1.00). For the general population in non-psychiatric cohort, the incidence of total NPAEs was much higher, 75.3% and 78.5% for varenicline (1427) and NRT (1200) groups, respectively (aOR 0.82, 95% CI 0.68 to 0.99). For the COPD population (1958), there were no differences in the incidence of NPAEs between comparison groups in both the psychiatric cohort (aOR 0.97, 95% CI 0.66 to 1.44) and non-psychiatric cohort (aOR 0.81, 95% CI 0.54 to 1.20). Results from subgroup or sensitivity analyses also did not reveal increased risks of NPAEs, but showed decreased risk of some subgroup NPAEs associated with varenicline.

Conclusions: In contrast to the concerns of a possible increased risk of NPAEs among varenicline users, we found a relative decreased risk of total NPAEs in varenicline users of the general population in psychiatric or non-psychiatric cohorts compared with NRT and no difference for NPAEs between varenicline and NRT users in smaller population with COPD.

For peer review only - http://bmjopen.bmj.com/site/about/guidelines.xhtml

Strengths and Limitations of This Study

- The use of large real-world population data makes the findings from this study more applicable to daily clinical practice.
- The risk of neuropsychiatric adverse events associated with varenicline were explored in both the general population and potential COPD patients.
- Subgroup and sensitivity analysis were performed to confirm the robustness and generalizability of the study findings.
- Despite the control for potential measured confounding and stratification according to risk groups, unmeasured healthy user bias may still be present, but is unlikely to mask substantial risks of varenicline.
- Behavioral changes like self-harm or suicide could not be evaluated in this study.

For peer review only

Introduction

Tobacco smoking is the leading preventable risk factor for a range of physical and mental illnesses,[1-4] which poses enormous threats to global public health.[5] Although average global smoking rates have declined since 1990 through tobacco control policies,[6] the actual number of smokers and disease burden related to smoking continues to increase owing to population growth.[7] More than 8 million people are killed by tobacco use each year.[8] Therefore, more intensified efforts are needed to fight this deadly epidemic. Smoking cessation strategies as key interventions to prevent smoking-related diseases are therefore urgently needed.[9] Varenicline was the first-line non-nicotine pharmacotherapy for smoking cessation and has greater efficacy than bupropion, single form nicotine replacement therapy (NRT) or placebo.[10,11] However, substantial concerns regarding its neuropsychiatric safety (e.g. suicidal thoughts, aggressive behavior) have been raised by case-reports since its approval in the United States in 2006.[12] Therefore, after the first safety communication and public health advisory in 2008, the U.S. Food and Drug Administration (FDA) released a black box warning on July 1, 2009.[13] Of note, the early safety concerns regarding varenicline were from uncontrolled case reports, which could not establish the causality because of a lack of control or comparator. Afterwards, many randomized controlled trials (RCTs) were conducted to evaluate the possible risk of neuropsychiatric adverse events (NPAEs). Notably, pooled evidence of these RCTs did not indicate an association between varenicline and NPAEs.[14] Neither did the Evaluating Adverse Events in a Global Smoking Cessation Study (EAGLES) show a significant increase in NPAEs with varenicline relative to NRT or placebo.[10]

Although the Black Box warning in varenicline's product labelling was already removed by both FDA and European Medicines Agency (EMA) in 2016, there are still concerns among patients and physicians about its neuropsychiatric safety resulting from the limited external validity of existing evidence from RCTs, [15] especially for high risk population with increased smoking prevalence, such as those with COPD and psychiatric disorders.[16] Importantly, COPD patients are older and suffer from many comorbidities making these patients more susceptible to drug-drug interactions potentially leading to related adverse drug events (ADEs).[17] Similarly, it has been reported that individuals with psychiatric disorders are prone to experience relapse of psychiatric symptoms.[18,19] Although many RCTs and post

For peer review only - http://bmjopen.bmj.com/site/about/guidelines.xhtml

hoc analyses of some RCTs showed consistent results about varenicline safety in subjects with specific psychiatric disorder,[20-27] real-world varenicline safety among these high-risk population is only explored in few studies,[28,29] several related large observational studies of high-quality were all about general population, rather than specific high-risk population.[30-33] It is important to further investigate the psychiatric safety of varenicline in special patients with COPD or psychiatric disorder or both in real-world setting to strengthen the knowledge of varenicline safety.

We therefore conducted this cohort study based on real-life data to assess the risk of NPAEs in starters with varenicline versus NRT starters in both the general and the COPD population with and without psychiatric disorders.

For peer review only

Methods

Study design and setting

We conducted a retrospective cohort study based on the University of Groningen pharmacy dispensing database IADB.nl (http://www.iadb.nl/) which has been widely used for various drug utilization studies.[34] IADB.nl contains information of prescribed medications from 70 community pharmacies covering a representative population of approximately 700,000 persons of the Netherlands, regardless of insurance type. It provides both patient information (e.g., date of birth, gender) and complete prescription records including the date of dispensing, quantity dispensed, dose regimen, the number of days the drug will be used, and the related Anatomical Therapeutic Chemical (ATC) code. The ATC code is a unique code assigned to a medicine according to the organ or system it works on and how it works. The ATC classification system proposed by the World Health Organization is a widely accepted drug classification scheme.

Study population

We included adult patients (>18 years) who started with varenicline or NRT. The individuals only prescribed varenicline (ATC code: N07BA03) or NRT (N07BA01) were included as anti-smoking drug users from the general population. Individuals that were prescribed drugs for obstructive airway disease (R03) at least 3 times within 1 year since 1st prescription after the age of 40 years were defined as COPD

For peer review only - http://bmjopen.bmj.com/site/about/guidelines.xhtml

patients, COPD patients who were prescribed varenicline or NRT were included as COPD anti-smoking drug users.

For both the general and COPD population with anti-smoking drug use, the first prescription date of varenicline and NRT was set as entry date (index date) of participants for exposure and control groups, respectively. To exclude the influence of other smoking cessation drugs on NPAEs, those who were prescribed other smoking cessation drugs including bupropion (N06AX12), nortriptyline (N06AA10) and cytisine (N07BA04) rather than the studied drugs (varenicline, NRT) within 180 days before or 180 days after index date were excluded. In order to get enough prescription records to define comorbidities and NPAEs, those who were registered in IADB.nl less than 6 months before or after index date were also excluded. For individuals who were prescribed both varenicline and NRT and met criteria of both groups (see Figure 1), we allocated the study subject to the group with the first index date according to the intention-to-treat principle.

In both the general and the COPD population, we classified the individuals into a psychiatric cohort and non-psychiatric cohort according to the presence of psychiatric disorders defined by the prescription of two or more drugs of analgesics (ACT codes N02, see supplementary Table S1), antiepileptics (N03), anti-parkinson drugs (N04), psycholeptics (N05) or psychoanaleptics (N06) within 6 months before index. In all, our study population covered four separate cohorts (1. General population in psychiatric cohort, 2. General population in non-psychiatric cohort, 3. COPD population in psychiatric cohort and 4. COPD population in non-psychiatric cohort) in which the association between exposure and outcomes were assessed (Figure 1).

Exposure and outcomes

We defined individuals using varenicline as the exposure group and those using NRT as the reference group. The primary outcome was incidence of any drug-treated neuropsychiatric adverse event (NPAE) including depression (ATC codes N06, antidepressants; N06CA, antidepressants in combination with psycholeptics), anxiety (N05B, anxiolytics) and insomnia (N05C, hypnotics and sedatives; see supplementary Table S1), defined as one or more new or naive prescriptions of the specified drugs mentioned above within 24 weeks after index date. Considering the use of varenicline could be possibly

For peer review only

For peer review only - http://bmjopen.bmj.com/site/about/guidelines.xhtml

extended to 24 weeks, which is also a suggested observed period by previous related studies.[28] Those who used the drugs under above-mentioned ATC codes level for NPAEs that prescribed before index date but without new prescription after index date were not count as people with NPAE. Those who never be prescribed drugs under above-mentioned ATC codes level before index date, but were prescribed (naive prescription) after index date will be included as people with NPAE.

Covariates

The following covariates were included as possible confounders: age, gender, social economic status (SES) based on postal codes, previous psychiatric disorders and other comorbidities including heart failure, ischemic heart disease, hypertension, cancer, diabetes, osteoporosis, peptic ulcer and gastroesophageal reflux disease (GERD), rheumatic arthritis, thyroid disorder, anemia, glaucoma, gout, and allergic rhinitis. All the comorbidities were defined by at least two prescriptions of related drugs within 6 months prior to index date (see detailed ATC codes in supplementary Table S1), The ATC codes used to define the comorbidities in this study were consistent with those in previous published papers.[35-39]

Statistical methods

The continuous and categorical variables are presented as means with standard deviations (SD) and numbers with percentages, respectively. The differences of characteristics between two groups were compared using student's t-tests and chi-square tests for continuous and categorical variables, respectively. Binary logistic regression modeling was used to obtain the odds ratio (OR) and corresponding 95% confidence interval (CI) after adjustment for the potential confounders. A two-sided $p \leq 0.05$ was considered to be statistically significant for all tests. All analyses were performed using IBM SPSS statistics 25 (IBM Corporation, Armonk, NY, USA) for Windows.

Subgroup and sensitivity analyses

Considering the possible influence of age and gender on the association between NPAEs and varenicline use, we further analyzed the primary outcome in the four cohorts (general and COPD populations with or without psychiatric disease, separately) by stratifying the results by age groups and gender. To further

For peer review only

For peer review only - http://bmjopen.bmj.com/site/about/guidelines.xhtml

test the robustness of the results, we performed several sensitivity analyses. First, because the possible treatment duration of varenicline is just 12 weeks, we explored the outcome occurring within 12 weeks after treatment initiation. Second, to exclude an active psychiatric status with possible frequently medication change, we further limit the study population only to those subjects who were not prescribed any drugs for psychiatric disorders within 30 days before index date. Thirdly, considering the influence of policy changes about reimbursement of smoking cessation treatment on the use of anti-smoking drugs,[40] we also performed a sensitivity analysis by excluding the patients whose prescription date of varenicline or NRT may be in the period of Dutch smoking policy changes (i.e. from July 1st 2011 to June 30, 2013). Finally, to exclude the possible selection bias for treatments, we also apply the inverse probability of treatment weighted (IPTW) regression to further test the robustness of our main results.

Patient and public involvement

Patients were not involved in the design or conduction of the study, as this research was based on established cohort and previously collected data. The results of this study will be distributed in scientific reports or academic conferences to benefit policy-makers, clinicians and patients.

Results

Baseline characteristics

In total, we included 9077 subjects who initiated varenicline or NRT from the general population, of which 2627 had psychiatric disorders (varenicline vs NRT: 1427 vs 1200), while 6450 did not have psychiatric disorder (varenicline vs NRT: 4480 vs 1970). For the COPD population, we included 1598 individuals, of which 649 had psychiatric disorders (varenicline vs NRT: 327 vs 322), 949 did not have psychiatric disorders (varenicline vs NRT: 608 vs 341). In both the general and COPD population, individuals treated with varenicline were younger than those treated with NRT (Table 1). Drug use for heart failure and ischemic heart disease was lower in the varenicline-treated than the NRT-treated group. In patients without psychiatric disorders, drug use for other comorbidities (e.g. diabetes and osteoporosis) was also less in individuals treated with varenicline than in those with NRT.

For peer review only - http://bmjopen.bmj.com/site/about/guidelines.xhtml

Primary outcome in the general and COPD population

In the general population with psychiatric disorders, the incidence of overall NPAEs within 24 weeks was lower in the varenicline group than the NRT group (75.3% vs 78.5%, OR 0.83 [0.69, 1.00], Table 2). After adjusting for potential confounders, the association did not substantially change (adjusted OR (aOR) 0.82, 95% CI [0.68, 0.99]). All the specific NPAEs were also less in the varenicline group than the NRT group, although the difference between the two comparison groups for depression and insomnia events did not reach statistical significance.

In the general population without psychiatric disorders, the incidence rates of NPAEs were lower than among those with psychiatric disorders. The incidence of overall NPAEs within 24-weeks in the varenicline group was lower than those in the NRT group (10.5% and 12.6%, respectively; aOR 0.85, 95% CI [0.72, 1.00]). No difference was observed between the two treatment groups for depression and anxiety, however, less insomnia was seen for varenicline than NRT (aOR was 0.63, 95% CI [0.49, 0.82]).

In the COPD population, we did not see a statistically significant difference for incidence of overall NPAEs between the varenicline and NRT groups for both the psychiatric cohort (aOR 0.97, 95% CI [0.66, 1.44], Table 3) and the non-psychiatric cohort (aOR 0.81, 95% CI [0.54, 1.20]). There were also no differences for specific NPAEs between treatment groups in these two cohorts except for anxiety, which was observed significantly less in the varenicline group compared with the NRT group (aOR 0.68, 95% CI [0.49, 0.94] for the psychiatric cohort only.

In the model for the primary outcome of NPAEs among overall general population, there were no significant interaction between treatments (varenicline vs NRTs) and cohorts (psychiatric vs non-psychiatric) interaction, which is also the same for the model of NPAEs among overall COPD patients.

Subgroup analysis

In the general population with psychiatric disorders, the risk of overall NPAEs was even lower among varenicline than NRT users for younger patients (aOR 0.54, 95% CI [0.28, 1.04] for age < 40, aOR 0.78, 95% CI [0.63, 0.98] for age 40-65, supplementary Table S2) and females (aOR 0.74, 95% CI [0.57, 0.97]). However, in the general population without psychiatric disorders, a lower risk of overall NPAE

For peer review only - http://bmjopen.bmj.com/site/about/guidelines.xhtml

by varenicline treatment compared with NRT was seen in older patients (aOR 0.53, 95% CI [0.34, 0.83]) and male subjects (aOR 0.78, 95% CI [0.61, 0.99]). In the COPD population, in both the psychiatric and non-psychiatric cohorts, there was no difference for overall NPAEs between the varenicline and NRT groups in each age group and gender group (supplementary Table S2).

Sensitivity analysis

In the general population, considering a follow-up of 12 weeks, the risk of overall NPAEs was less for varenicline compared with NRT in both the psychiatric and non-psychiatric cohorts (aOR 0.78, 95% CI: 0.64 to 0.94; aOR 0.74, 95% CI: 0.62 to 0.89; respectively, supplementary Table S3). After limiting the study population to those who were not prescribed drugs for any psychiatric disorder or those who were not prescribed drugs for depression, anxiety and insomnia within 1 month before index date, there was no statistically significant difference for overall NPAEs, irrespective of presence of psychiatric disorders in the previous year (supplementary Table S4). The result was similar when we limited our cohort to individuals whose study period was not in the period of Dutch smoking policy change; the aOR for varenicline compared with NRT was 0.86, 95% CI [0.69, 1.07] in the psychiatric cohort and 0.86, 95% CI [0.71, 1.03] in non-psychiatric cohort.

In the COPD population, there were no statistically significant differences for overall and subgroup NPAEs within follow-up of 12 weeks between the two treatments except for anxiety in the psychiatric cohort (aOR 0.64, 95% CI [0.46, 0.90], supplementary Table S5). After limiting the study population to those who were not prescribed drugs for any psychiatric disorder or those who were not prescribed drugs for depression, anxiety and insomnia within 1 month before index date, there was no statistically significant difference for overall NPAEs, irrespective of presence of psychiatric disorders (supplementary Table S6). Similar results were seen when limiting individuals to those whose study period was not in the period of Dutch smoking policy change (OR 0.97, 95% CI: 0.62 to 1.51; OR 1.00, 95% CI: 0.64 to 1.58; respectively for the psychiatric and non-psychiatric cohorts, supplementary Table S6).

After adjusting the results by IPTW regression in cohorts of general populations, the results (supplementary Table S4) are consistent with the results presented above. Also, no significant change

For peer review only - http://bmjopen.bmj.com/site/about/guidelines.xhtml

(supplementary Table S6) was observed by IPTW regression for cohorts of COPD patients compared with results above.

Discussion

Main findings and interpretation

Within 24 weeks following initiation of varenicline treatment, we found no significantly increased risk of NPAEs in both the general and COPD population compared with those using NRT, irrespective of the presence of psychiatric disorders. These findings are consistent with the results of previous RCTs and large observational studies,[10,41,42] Considering the fact that the smoking cessation treatment may last for only 12 weeks without further treatment,[43] we also explored the NPAEs in this shorter time period and observed no increased risk in overall and specific NPAEs for varenicline compared with NRT.

In contrast to the concerns about a possible increased risk of NPAEs among varenicline users, we found an 18% and 15% relative decrease in NPAEs in varenicline users of the general population with and without psychiatric disorders, respectively, compared with NRT. Regarding the safety of varenicline for specific NPAEs, we recorded a 29% reduced risk of anxiety by varenicline (vs NRT) in the psychiatric cohort, and a 37% reduced risk of insomnia in the non-psychiatric cohort. Rates of depression events were comparable between the two groups in both psychiatric and non-psychiatric cohorts among the general population. These results were consistent with the pooled results of 39 RCTs in a meta-analysis,[14] which indicated that less anxiety (hazard ratio (HR) 0.75, 95% CI: 0.61, 0.93) was also observed in the varenicline group (vs NRT), and depression episodes were also evenly distributed among two treatments (HR 0.96, 95% CI: 0.75, 1.22). Compared with our study, the difference is that in this review an increased risk of insomnia was observed in the varenicline group (HR 1.56, 95% CI: 1.36, 1.78).[14] Of note, the aforementioned review did not explore the risk of varenicline on NPAEs separately in those with and without psychiatric disorders, which may contribute to the observed differences in this review compared to our results. Of note, our result is consistent with another cohort study based data from the general practice that a declined depression were observed to be associated with varenicline (HR 0.88 [0.77-1.00]),[30] similar finding was also reported in a clinical trial.[44]

For peer review only - http://bmjopen.bmj.com/site/about/guidelines.xhtml

COPD patients are considered a high-risk population with high prevalence of smoking and relatively older age, making these persons more susceptible for possible adverse drug reactions (ADEs).[17,45] Of note, in our study we did not observe an increased risk of overall NPAEs among COPD patients using varenicline in both the psychiatric and non-psychiatric cohorts. Of note, regarding the occurrence of specific NPAEs, less anxiety was seen in the varenicline group than in the NRT group in both cohorts. The safety of varenicline was not fully explored among COPD patients in previous studies. To the best of our knowledge, only two studies (one RCT and one cohort study) were previously conducted.[28,46] Similar to our results, both of these two studies did not find an increased risk of NPAEs for varenicline. Notably, in the cohort study even a reduced risk of depression was observed in varenicline users compared with users of NRT among COPD patients.[28] This may be misled by unmeasured confounders, however, after modelling the effects of possible unmeasured confounders, the author concluded that an increased risk of these adverse events was very unlikely.[28]

It is notable that there is a large heterogeneity in the definition of NPAEs across studies. In some studies, the investigators focused on moderate to serious adverse events like depression, suicide or mental disorders that require hospitalization or an emergency department visit.[28,32,42,47] While other studies included all adverse symptoms (e.g. angry, nervousness) or adverse events such as traffic offences.[10,43] In this study, we used prescriptions to define neuropsychiatric outcomes for the most commonly reported NPAEs including depression, anxiety and insomnia during the study period. Despite differences in clinical definitions, the observed 24-week event rates of specific NPAEs (3%-9%) for depression, anxiety or insomnia in our general population without psychiatric disorders were similar to previous studies.[14] However, the rate of NPAEs was substantially higher among participants in psychiatric cohort than those in non-psychiatric cohort, which was also consistent with findings from previous studies.[10,20,21,48] When we further limited our study population to those who did not experience any psychiatric disorder or not experienced any depression, anxiety and insomnia within 1 month of enrollment, we found that both the overall NPAEs and specific NPAE reduced substantially.

Although the rates of NPAEs are different between psychiatric and non-psychiatric cohorts in this study, the presence of psychiatric disorders did not influence the risk of NPAEs by varenicline compared with

For peer review only - http://bmjopen.bmj.com/site/about/guidelines.xhtml

NRT which was also consistent with previous studies.[10,49] In a prospective longitudinal study among psychiatric patients, there was no exacerbation of psychiatric symptoms detected except gastrointestinal adverse events.[50] However, although it is not within the scope of this study, what need to be mentioned is that an increased rate of outpatient visits for schizophrenia was previously reported to be present only in patients with a pre-existing mental health disorder.[32] This may be explained by mediation through individual genetic liability.[51]

In interpreting the absence of increased risks by varenicline (vs NRT) observed in the general population, we can only speculate that the positive effect from varenicline may result from its effect of consistently reducing withdrawal-related symptoms of negative affect and raised levels of positive affect.[52] It has been reported that varenicline yields higher abstinence rates than NRT,[10,11] irrespective of smoker characteristics.[53] The successful quitting of smoking by varenicline may offer more benefits to the psychiatric status compared with NRT. Moreover, there is evidence that quitting smoking is associated with recovery in stress, anxiety and depression in smokers.[44,54,55] A significant and progressive improvement of anxiety and depression was also reported in an observational study, and the protective effect was observed regardless of the presence of psychiatric pathology.[49]

Many studies demonstrated gender differences in varenicline efficacy for smoking cessation,[56,57] some studies also found a difference in neuropsychiatric events between genders.[58] In this study, we found less risk of NPAEs by varenicline for females in the psychiatric cohort compared with NRT. This may be explained by the better therapeutic response to varenicline in women compared with men. [56] However, in the non-psychiatric cohort, we observed less risk of NPAEs in males by varenicline (vs NRT) which we cannot explain and more research needs to be done on gender disparities. Similarly, there are some indications for age group-dependent NPAEs risk by varenicline (vs NRT) in two cohorts of this study. In the psychiatric cohort, there were lower event rates in the varenicline group than the NRT group in younger age groups. Contrary, in the non-psychiatric cohort, lower event rates were seen in older users of varenicline than NRT. The age-specific effectiveness of varenicline relative to NRT patch or gum were also reported that only younger smokers achieved greater likelihood of abstinence than NRTs.[59]As such, age disparities also need to be studied more closely.

For peer review only - http://bmjopen.bmj.com/site/about/guidelines.xhtml

Strengths and limitations

A major strength of this study is that we evaluated the safety of varenicline in both the general and COPD population based on large real-life population data making the results representative and more applicable to daily clinical practice. Both short- (12 weeks) and long-term (24 weeks) NPAEs after treatment initiation were explored in this study. Besides the influence of current or previous psychiatric disorders, we also evaluated the influence of age and gender on the NPAEs between treatment groups. We excluded the influence of a psychiatric disorder status on the outcomes in the two comparison groups by classifying the population into two cohorts (psychiatric cohort and non-psychiatric cohort) in the stage of study design. Additionally, to test the robustness of our study results, sensitivity analyses and propensity score analysis were conducted.

Like the limitations mentioned in two previous related observational studies,[41,42] our study also has the issue of potential of unmeasured healthy user bias, although we tried our best to exclude the influence of measured baseline differences between exposure groups by adjusting for them. These unmeasured confounders could be controlled by some novel methods such as instrumental variable analysis; however, this analysis is not available in this study due to lack of a proper instrumental variable. Second, as outcome events and comorbidities were defined by prescriptions of related drugs as proxies, which is a crude measure and may have led to some misclassification. Besides, the prescription of medication may not always lead to actually intake of the drugs (non-adherence) and such misclassification, if random, may have caused associations to be biased towards the null value. Third, some serious behavioral changes like self-harm or suicide and minor symptoms could not be evaluated in this study due to the limitation of the prescription database, thus we only included the most frequently reported NPAEs that are commonly drug treated. Lastly, SES was determined by the postal code level, which could lead to some misclassification, although postal code was a frequently used predictor of SES.[60]

Conclusion

From this population-based real-life cohort study, we conclude that varenicline is unlikely associated with an increased risk of NPAEs in both general and COPD patients with or without psychiatric

For peer review only - http://bmjopen.bmj.com/site/about/guidelines.xhtml

disorders following its initiation compared with NRT. Of note, varenicline were even associated with lower risk of some subgroup NPAEs than NRTs in sub-cohorts of our population. Our findings are notably and consistent with previous literature. These results provide further support for the safety of varenicline to quit smoking in both the general and COPD populations.

For peer review only

For peer review only - http://bmjopen.bmj.com/site/about/guidelines.xhtml

Acknowledgement

The authors would like to thank IADB.nl pharmacies for generous contributions of providing their data for research.

Contributions

YW and EH are involved in designing the study, JHB contributed the data collection, YW analyzed the data and drafted the article, CCMSV, HMB, JFMB, BW, EH contributed to the interpretation of results and revision of the manuscript. All authors read and approved the final manuscript.

Funding

This study was supported by internal funding. YW got the funding from China Scholarship Council (file no: 201506010259) for her PhD study. The funders had no role in study design, data collection and analysis, decision to publish, or preparation of the manuscript.

Competing interests

None declared.

Data sharing statement

Data are available upon reasonable request.

Ethics approval

This study is based on established database IADB.nl, data are collected in accordance with the national and European guidelines on privacy requirements for handling human data. The authors have no ethical conflicts to disclose. Ethics approval is not needed and required for this study.

Supplementary data

Table S1 to S6 are available as Supplementary data.

For peer review only - http://bmjopen.bmj.com/site/about/guidelines.xhtml

References

1. Banks E, Joshy G, Korda RJ, et al. Tobacco smoking and risk of 36 cardiovascular disease subtypes: fatal and non-fatal outcomes in a large prospective Australian study. *BMC Med.* 2019;17(1):128.

2. Gaudet MM, Carter BD, Brinton LA, et al. Pooled analysis of active cigarette smoking and invasive breast cancer risk in 14 cohort studies. *Int J Epidemiol.* 2017;46(3):881-893.

3. Tjora T, Hetland J, Aaro LE, Wold B, Wiium N, Overland S. The association between smoking and depression from adolescence to adulthood. *Addiction.* 2014;109(6):1022-1030.

4. Evins AE, Korhonen T, Kinnunen TH, Kaprio J. Prospective association between tobacco smoking and death by suicide: a competing risks hazard analysis in a large twin cohort with 35-year follow-up. *Psychol Med.* 2017;47(12):2143-2154.

5. Thun MJ, Carter BD, Feskanich D, et al. 50-Year Trends in Smoking-Related Mortality in the United States. *New Engl J Med.* 2013;368(4):351-364.

6. Collaborators GBDT. Smoking prevalence and attributable disease burden in 195 countries and territories, 1990-2015: a systematic analysis from the Global Burden of Disease Study 2015. *Lancet.* 2017;389(10082):1885-1906.

7. The L. Progress towards a tobacco-free world. *Lancet.* 2018;392(10141):1.

8. World Health Organisation. WHO report on the global tobacco epidemic 2019: offer help to quit tobacco use. Geneva: World Health Organisation, 2019: 17-21.

9. Mehrotra R, Yadav A, Sinha DN, et al. Smokeless tobacco control in 180 countries across the globe: call to action for full implementation of WHO FCTC measures. *Lancet Oncol.* 2019;20(4):e208-e217.

10. Anthenelli RM, Benowitz NL, West R, et al. Neuropsychiatric safety and efficacy of varenicline, bupropion, and nicotine patch in smokers with and without psychiatric disorders (EAGLES): a double-blind, randomised, placebo-controlled clinical trial. *Lancet.* 2016;387(10037):2507-2520.

11. Ebbert JO, Hughes JR, West RJ, et al. Effect of varenicline on smoking cessation through smoking reduction: a randomized clinical trial. *JAMA.* 2015;313(7):687-694.

12. Moore TJ, Furberg CD, Glenmullen J, Maltsberger JT, Singh S. Suicidal behavior and depression in smoking cessation treatments. *PLoS One.* 2011;6(11):e27016.

13. US Food and Drug Administration. Public health advisory: FDA requires new boxed warnings for the smoking cessation drugs Chantix and Zyban. http://wayback.archive-it.org/7993/20170112005513/http://www.fda.gov/Drugs/DrugSafety/PostmarketDrugSafetyInformationforPatientsandProviders/ucm169988.htm. Published July 1, 2009. Accessed June 7, 2019. .

14. Thomas KH, Martin RM, Knipe DW, Higgins JP, Gunnell D. Risk of neuropsychiatric adverse events associated with varenicline: systematic review and meta-analysis. *BMJ.* 2015;350:h1109.

15. Rothwell PM. External validity of randomised controlled trials: "to whom do the results of this trial apply?". *Lancet.* 2005;365(9453):82-93.

16. Lawrence D, Mitrou F, Zubrick SR. Smoking and mental illness: results from population surveys in Australia and the United States. *BMC Public Health.* 2009;9:285.

17. Wang Y, Bahar MA, Jansen AME, et al. Improving antibacterial prescribing safety in the management of COPD exacerbations: systematic review of observational and clinical studies

For peer review only - http://bmjopen.bmj.com/site/about/guidelines.xhtml

on potential drug interactions associated with frequently prescribed antibacterials among COPD patients. *J Antimicrob Chemother.* 2019;74(10):2848-2864.

18. Garza D, Murphy M, Tseng LJ, Riordan HJ, Chatterjee A. A double-blind randomized placebo-controlled pilot study of neuropsychiatric adverse events in abstinent smokers treated with varenicline or placebo. *Biol Psychiatry.* 2011;69(11):1075-1082.

19. Tonstad S, Davies S, Flammer M. Psychiatric Adverse Events in Randomized, Double-Blind, Placebo-Controlled Clinical Trials of Varenicline: A Pooled Analysis (vol 33, pg 298, 2010). *Drug Safety.* 2010;33(6):522-522.

20. Heffner JL, Evins AE, Russ C, et al. Safety and efficacy of first-line smoking cessation pharmacotherapies in bipolar disorders: Subgroup analysis of a randomized clinical trial. *J Affect Disord.* 2019;256:267-277.

21. Evins AE, West R, Benowitz NL, et al. Efficacy and Safety of Pharmacotherapeutic Smoking Cessation Aids in Schizophrenia Spectrum Disorders: Subgroup Analysis of EAGLES. *Psychiatr Serv.* 2020:appips202000032.

22. Chengappa KN, Perkins KA, Brar JS, et al. Varenicline for smoking cessation in bipolar disorder: a randomized, double-blind, placebo-controlled study. *J Clin Psychiatry.* 2014;75(7):765-772.

23. Evins AE, Cather C, Pratt SA, et al. Maintenance treatment with varenicline for smoking cessation in patients with schizophrenia and bipolar disorder: a randomized clinical trial. *JAMA.* 2014;311(2):145-154.

24. Williams JM, Anthenelli RM, Morris CD, et al. A randomized, double-blind, placebo-controlled study evaluating the safety and efficacy of varenicline for smoking cessation in patients with schizophrenia or schizoaffective disorder. *J Clin Psychiatry.* 2012;73(5):654-660.

25. Pachas GN, Cather C, Pratt SA, et al. Varenicline for Smoking Cessation in Schizophrenia: Safety and Effectiveness in a 12-Week, Open-Label Trial. *J Dual Diagn.* 2012;8(2):117-125.

26. Anthenelli RM, Morris C, Ramey TS, et al. Effects of varenicline on smoking cessation in adults with stably treated current or past major depression: a randomized trial. *Ann Intern Med.* 2013;159(6):390-400.

27. Evins AE, Benowitz NL, West R, et al. Neuropsychiatric Safety and Efficacy of Varenicline, Bupropion, and Nicotine Patch in Smokers With Psychotic, Anxiety, and Mood Disorders in the EAGLES Trial. *J Clin Psychopharmacol.* 2019;39(2):108-116.

28. Kotz D, Viechtbauer W, Simpson CR, van Schayck OCP, West R, Sheikh A. Cardiovascular and neuropsychiatric risks of varenicline and bupropion in smokers with chronic obstructive pulmonary disease. *Thorax.* 2017;72(10):905-911.

29. Taylor GMJ, Itani T, Thomas KH, et al. Prescribing Prevalence, Effectiveness, and Mental Health Safety of Smoking Cessation Medicines in Patients With Mental Disorders. *Nicotine Tob Res.* 2020;22(1):48-57.

30. Gunnell D, Irvine D, Wise L, Davies C, Martin RM. Varenicline and suicidal behaviour: a cohort study based on data from the General Practice Research Database. *BMJ.* 2009;339:b3805.

31. Meyer TE, Taylor LG, Xie S, et al. Neuropsychiatric events in varenicline and nicotine replacement patch users in the Military Health System. *Addiction.* 2013;108(1):203-210.

32. Cunningham FE, Hur K, Dong D, et al. A comparison of neuropsychiatric adverse events during early treatment with varenicline or a nicotine patch. *Addiction.* 2016;111(7):1283-1292.

33. Gibbons RD, Mann JJ. Varenicline, smoking cessation, and neuropsychiatric adverse events. *Am J Psychiatry.* 2013;170(12):1460-1467.

For peer review only - http://bmjopen.bmj.com/site/about/guidelines.xhtml

34. Visser ST, Schuiling-Veninga CC, Bos JH, de Jong-van den Berg LT, Postma MJ. The population-based prescription database IADB.nl: its development, usefulness in outcomes research and challenges. *Expert Rev Pharmacoecon Outcomes Res.* 2013;13(3):285-292.

35. Kronig M, Haverkamp C, Schulte A, et al. Diabetes and beta-adrenergic blockage are risk factors for metastatic prostate cancer. *World J Surg Oncol.* 2017;15(1):50.

36. van Boven JF, van Raaij JJ, van der Galien R, et al. Impact of multiple-dose versus single-dose inhaler devices on COPD patients' persistence with long-acting beta(2)-agonists: a dispensing database analysis. *NPJ Prim Care Respir Med.* 2014;24:14069.

37. Huber CA, Szucs TD, Rapold R, Reich O. Identifying patients with chronic conditions using pharmacy data in Switzerland: an updated mapping approach to the classification of medications. *Bmc Public Health.* 2013;13.

38. Monte S, Macchia A, Romero M, D'Ettorre A, Giuliani R, Tognoni G. Antidepressants and cardiovascular outcomes in patients without known cardiovascular risk. *Eur J Clin Pharmacol.* 2009;65(11):1131-1138.

39. Chini F, Pezzotti P, Orzella L, Borgia P, Guasticchi G. Can we use the pharmacy data to estimate the prevalence of chronic conditions? a comparison of multiple data sources. *Bmc Public Health.* 2011;11.

40. van Boven JF, Vemer P. Higher Adherence During Reimbursement of Pharmacological Smoking Cessation Treatments. *Nicotine Tob Res.* 2016;18(1):56-63.

41. Kotz D, Viechtbauer W, Simpson C, van Schayck OC, West R, Sheikh A. Cardiovascular and neuropsychiatric risks of varenicline: a retrospective cohort study. *Lancet Respir Med.* 2015;3(10):761-768.

42. Thomas KH, Martin RM, Davies NM, Metcalfe C, Windmeijer F, Gunnell D. Smoking cessation treatment and risk of depression, suicide, and self harm in the Clinical Practice Research Datalink: prospective cohort study. *BMJ.* 2013;347:f5704.

43. Molero Y, Lichtenstein P, Zetterqvist J, Gumpert CH, Fazel S. Varenicline and risk of psychiatric conditions, suicidal behaviour, criminal offending, and transport accidents and offences: population based cohort study. *BMJ.* 2015;350:h2388.

44. Cather C, Hoeppner S, Pachas G, et al. Improved Depressive Symptoms in Adults with Schizophrenia During a Smoking Cessation Attempt with Varenicline and Behavioral Therapy. *J Dual Diagn.* 2017;13(3):168-178.

45. Mangoni AA, Jackson SH. Age-related changes in pharmacokinetics and pharmacodynamics: basic principles and practical applications. *Br J Clin Pharmacol.* 2004;57(1):6-14.

46. Tashkin DP, Rennard S, Hays JT, Ma W, Lawrence D, Lee TC. Effects of varenicline on smoking cessation in patients with mild to moderate COPD: a randomized controlled trial. *Chest.* 2011;139(3):591-599.

47. Pasternak B, Svanstrom H, Hviid A. Use of varenicline versus bupropion and risk of psychiatric adverse events. *Addiction.* 2013;108(7):1336-1343.

48. Ayers CR, Heffner JL, Russ C, et al. Efficacy and safety of pharmacotherapies for smoking cessation in anxiety disorders: Subgroup analysis of the randomized, active- and placebo-controlled EAGLES trial. *Depress Anxiety.* 2020;37(3):247-260.

49. Pacheco VA, Fernandez APGB, Morales AV, Crespo EL, Garcia SM, Rubio TM. Anxiety, Depression and Tobacco Abstinence. *Adicciones.* 2017;29(4):233-244.

50. Raich A, Ballbe M, Nieva G, et al. Safety of Varenicline for Smoking Cessation in Psychiatric and Addicts Patients. *Subst Use Misuse.* 2016;51(5):649-657.

For peer review only - http://bmjopen.bmj.com/site/about/guidelines.xhtml

51. Agerbo E, Sullivan PF, Vilhjalmsson BJ, et al. Polygenic Risk Score, Parental Socioeconomic Status, Family History of Psychiatric Disorders, and the Risk for Schizophrenia: A Danish Population-Based Study and Meta-analysis. *JAMA Psychiatry.* 2015;72(7):635-641.

52. Global Strategy for the Diagnosis, Management and Prevention of COPD, Global Initiative for Chronic Obstructive Pulmonary Disease (GOLD) 2019 Report. Available from: http://goldcopd.org.

53. Walker N, Gainforth H, Kiparoglou V, Robinson H, van Woerden H, West R. Factors moderating the relative effectiveness of varenicline and nicotine replacement therapy in clients using smoking cessation services. *Addiction.* 2018;113(2):313-324.

54. Zarghami M, Taghizadeh F, Sharifpour A, Alipour A. Efficacy of Smoking Cessation on Stress, Anxiety, and Depression in Smokers with Chronic Obstructive Pulmonary Disease: A Randomized Controlled Clinical Trial. *Addict Health.* 2018;10(3):137-147.

55. Taylor G, McNeill A, Girling A, Farley A, Lindson-Hawley N, Aveyard P. Change in mental health after smoking cessation: systematic review and meta-analysis. *BMJ.* 2014;348:g1151.

56. McKee SA, Smith PH, Kaufman M, Mazure CM, Weinberger AH. Sex Differences in Varenicline Efficacy for Smoking Cessation: A Meta-Analysis. *Nicotine Tob Res.* 2016;18(5):1002-1011.

57. Smith PH, Kasza KA, Hyland A, et al. Gender differences in medication use and cigarette smoking cessation: results from the International Tobacco Control Four Country Survey. *Nicotine Tob Res.* 2015;17(4):463-472.

58. The Lancet P. Sex and gender in psychiatry. *Lancet Psychiatry.* 2016;3(11):999.

59. Chang PY, Shiu MN, Yuan YT, Chang HC, Su PY, Lan TH. Comparative Effectiveness of Varenicline and Nicotine Replacement Therapy for Smoking Cessation in Older and Younger Smokers: A Prospective Cohort in Taiwan. *Nicotine Tob Res.* 2019;21(2):149-155.

60. Gibson PH, Croal BL, Cuthbertson BH, et al. Socio-economic status and early outcome from coronary artery bypass grafting. *Heart.* 2009;95(10):793-798.

For peer review only

For peer review only - http://bmjopen.bmj.com/site/about/guidelines.xhtml

Tables and Figure

Table 1. Baseline characteristics of general and COPD population with and without psychiatric disorders by treatment groups.

Table 2. Incidence of neuropsychiatric adverse events (NPAEs) and association with varenicline compared with NRT in general populations with and without psychiatric disorders with follow up of 24 weeks.

Table 3. Incidence of neuropsychiatric adverse events (NPAEs) and association with varenicline compared with NRT in COPD population with and without psychiatric disorders within follow up of 24 weeks.

Figure 1. Flow chart of population selection. NRT: nicotine replacement therapies; ATC: anatomical therapeutic chemical.

For peer review only

For peer review only - http://bmjopen.bmj.com/site/about/guidelines.xhtml

Table 1. Baseline characteristics of general and COPD population with and without psychiatric disorders by treatment groups.

**Characteristics**	**General population (n = 9077)**				**COPD population (1598)**			
	Psychiatric cohort (n = 2627)		Non-psychiatric cohort (n = 6450)		Psychiatric cohort (n = 649)		Non-psychiatric cohort (n = 949)	
	Varenicline (N=1427)	NRT (N=1200)	Varenicline (N=4480)	NRT (N= 1970)	Varenicline (N=327)	NRT (N=322)	Varenicline (N=608)	NRT (N= 341)
**Age (years)**								
**Mean (SD)**	54.5 (9.6)*	55.8 (11.8)	52.1 (9.7)*	53.4 (12.5)	59.2 (8.6)*	62.4 (10.2)	58.9 (8.9)*	62.7 (9.8)
**Age range**	32 - 87	25 - 92	32 - 102	22 - 90	41 - 87	41 - 92	40 - 102	42 -89
**Gender (n, %)**								
**Men**	541 (37.9)*	523 (43.6)	2406 (53.7)	1100 (55.8)	111 (33.9)	126 (39.1)	310 (51.0)	184 (54.0)
**Female**	886 (62.1)	677 (56.4)	2074 (46.3)	870 (44.2)	216 (66.1)	196 (60.9)	298 (49.0)	157 (46.0)
**Year of index date (n, %)**								
**1994-2010**	519 (36.4)*	492 (41.0)	1497 (33.4)*	895 (45.4)	135 (41.3)	135 (41.9)	211 (34.7)	124 (36.4)
**2011-2017**	908 (63.6)	708 (59.0)	2983 (66.6)	1075 (54.6)	192 (58.7)	187 (58.1)	397 (65.3)	217 (63.6)
**Social economic status (n, %)**								
**Low**	714 (50.0)*	655 (54.6)	2117 (47.3)*	1080 (54.8)	170 (52.0)	172 (53.4)	287 (47.2)	175 (51.3)
**High**	713 (50.0)	545 (45.4)	2363 (52.7)	890 (45.2)	157 (48.0)	150 (46.6)	321 (52.8)	166 (48.7)
**Comorbidities (n, %)**								

For peer review only

For peer review only - http://bmjopen.bmj.com/site/about/guidelines.xhtml

**Heart failure**	41 (2.9)*	82 (6.8)	47 (1.0)*	53 (2.7)	23 (7.0)*	42 (13.0)	20 (3.3)*	27 (7.9)
**Ischemic heart disease**	25 (1.8)*	39 (3.3)	36 (0.8)*	31 (1.6)	9 (2.8)*	20 (6.2)	9 (1.5)*	12 (3.5)
**Hypertension**	508 (35.6)	465 (38.8)	1039 (23.2)*	547 (27.8)	146 (44.6)	167 (51.9)	234 (38.5)	145 (42.5)
**Cancers**	9 (0.6)*	0 (0.0)	9 (0.2)	9 (0.5)	0 (0.0)	0 (0.0)	0 (0.0)	2 (0.6)
**Diabetes mellitus**	162 (11.4)	129 (10.8)	260 (5.8)*	146 (7.4)	53 (16.2)	40 (12.4)	47 (7.7)*	43 (12.6)
**Osteoporosis**	39 (2.7)	32 (2.7)	25 (0.6)*	26 (1.3)	17 (5.2)	20 (6.2)	6 (1.0)*	14 (4.1)
**Peptic ulcer and GERD**	436 (30.6)	407 (33.9)	474 (10.6)*	253 (12.8)	134 (41.0)	142 (44.1)	123 (20.2)	82 (24.0)
**Rheumatic arthritis**	170 (11.9)	172 (14.3)	204 (4.6)*	123 (6.2)	45 (13.8)	41 (12.7)	40(6.6)	21 (6.2)
**Thyroid disorder**	55 (3.9)	40 (3.3)	96 (2.1)	49 (2.5)	18 (5.5)	13 (4.0)	18 (3.0)	5 (1.5)
**Anemia**	49 (3.4)	52 (4.3)	49 (1.1)*	38 (1.9)	11 (3.4)	20 (6.2)	4 (0.7)*	12 (3.5)
**Glaucoma**	13 (0.9)	12 (1.0)	17 (0.4)*	23 (1.2)	4 (1.2)	4 (1.2)	3 (0.5)*	7 (2.1)
**Gout**	4 (0.3)	9 (0.8)	16 (0.4)*	24 (1.2)	1 (0.3)	3 (0.9)	3 (0.5)*	10 (2.9)
**Allergic rhinitis**	76 (5.3)	47 (3.9)	104 (2.3)	34 (1.7)	28 (8.6)	24 (7.5)	33 (5.4)	15 (4.4)

*$p<0.05$; GERD: Gastroesophageal Reflux Disease. NRT: Nicotine replacement therapy.

For peer review only

For peer review only - http://bmjopen.bmj.com/site/about/guidelines.xhtml

Table 2. Incidence of neuropsychiatric adverse events (NPAEs) and association with varenicline compared with NRT in general population with and without psychiatric disorders with follow up of 24 weeks.

**NPAEs**	**Psychiatric cohort** Varenicline (n=1427) vs NRT (n=1200)			**Non-psychiatric cohort** Varenicline (n=4480) vs NRT (n=1970)		
	Events (n, %) #	Crude OR (95% CI)	Adjusted OR* (95% CI)	Events (n, %) #	Crude OR (95% CI)	Adjusted OR** (95% CI)
**Overall**	1074 (75.3): 942 (78.5)	0.83 [0.69, 1.00]	0.82 [0.68, 0.99]	469 (10.5): 248 (12.6)	0.81 [0.69, 0.96]	0.85 [0.72, 1.00]
Depression	629 (44.1): 548 (45.7)	0.94 [0.80, 1.09]	0.90 [0.77, 1.05]	148 (3.3): 57 (2.9)	1.15 [0.84, 1.56]	1.15 [0.84, 1.57]
Anxiety	456 (32.0): 472 (39.3)	0.72 [0.62, 0.85]	0.71 [0.61, 0.84]	215 (4.8): 110 (5.6)	0.85 [0.67, 1.08]	0.90 [0.71, 1.15]
Insomnia	372 (26.1): 352 (29.3)	0.85 [0.72, 1.01]	0.89 [0.75, 1.06]	147 (3.3): 105 (5.3)	0.60 [0.47, 0.78]	0.63 [0.49, 0.82]

NPAEs: neuropsychiatric adverse events, defined by new or naive prescription of related medications for depression, anxiety, or insomnia within 24 weeks after index date; NRT: nicotine replacement therapy; OR: odds ratio; CI: confidence interval; *Adjusted for age, gender, socioeconomic status, heart failure, ischemic heart disease and cancer. **Adjusted for age, socioeconomic status, heart failure, ischemic heart disease, hypertension, diabetes, osteoporosis, peptic ulcer and GERD, Rheumatic arthritis, anemia, glaucoma and gout; # Varenicline: NRT;

For peer review only - http://bmjopen.bmj.com/site/about/guidelines.xhtml

Table 3. Incidence of neuropsychiatric adverse events (NPAEs) and association with varenicline compared with NRT in COPD population with and without psychiatric disorders with follow up of 24 weeks.

NPAEs	**Psychiatric cohort** Varenicline (n=327) vs NRT (n=322)			**Non-psychiatric cohort** Varenicline (n=608) vs NRT (n=341)		
	Events (n, %) #	Crude OR (95% CI)	Adjusted OR* (95% CI)	Events (n, %) #	Crude OR (95% CI)	Adjusted OR** (95% CI)
**Overall**	258 (78.9): 260 (80.7)	0.89 [0.61, 1.31]	0.97 [0.66, 1.44]	76 (12.5): 53 (15.5)	0.78 [0.53, 1.13]	0.81 [0.54,1.20]
Depression	146 (44.6): 145 (45.0)	0.99 [0.72, 1.34]	0.90 [0.65, 1.24]	22 (3.6): 10 (2.9)	1.24 [0.58, 2.66]	1.37 [0.62, 3.03]
Anxiety	110 (33.6): 136 (42.2)	0.69 [0.50, 0.95]	0.68 [0.49, 0.94]	38 (6.3): 25 (7.3)	0.84 [0.50, 1.42]	0.86 [0.50, 1.49]
Insomnia	102 (31.2): 124 (38.5)	0.72 [0.52, 1.00]	0.84 [0.60, 1.18]	25 (4.1): 21 (6.2)	0.65 [0.36, 1.19]	0.63 [0.34, 1.17]

NPAEs: neuropsychiatric adverse events, defined by new or naive prescription of related medications for depression, anxiety or insomnia within 24 weeks after index date; NRT: nicotine replacement therapy; OR: odds ratio; CI: confidence interval; *Adjusted for age, heart failure, ischemic heart disease; **Adjusted for age, heart failure, ischemic heart disease, diabetes, osteoporosis, anemia, glaucoma and gout; # varenicline:NRT.

For peer review only

For peer review only - http://bmjopen.bmj.com/site/about/guidelines.xhtml

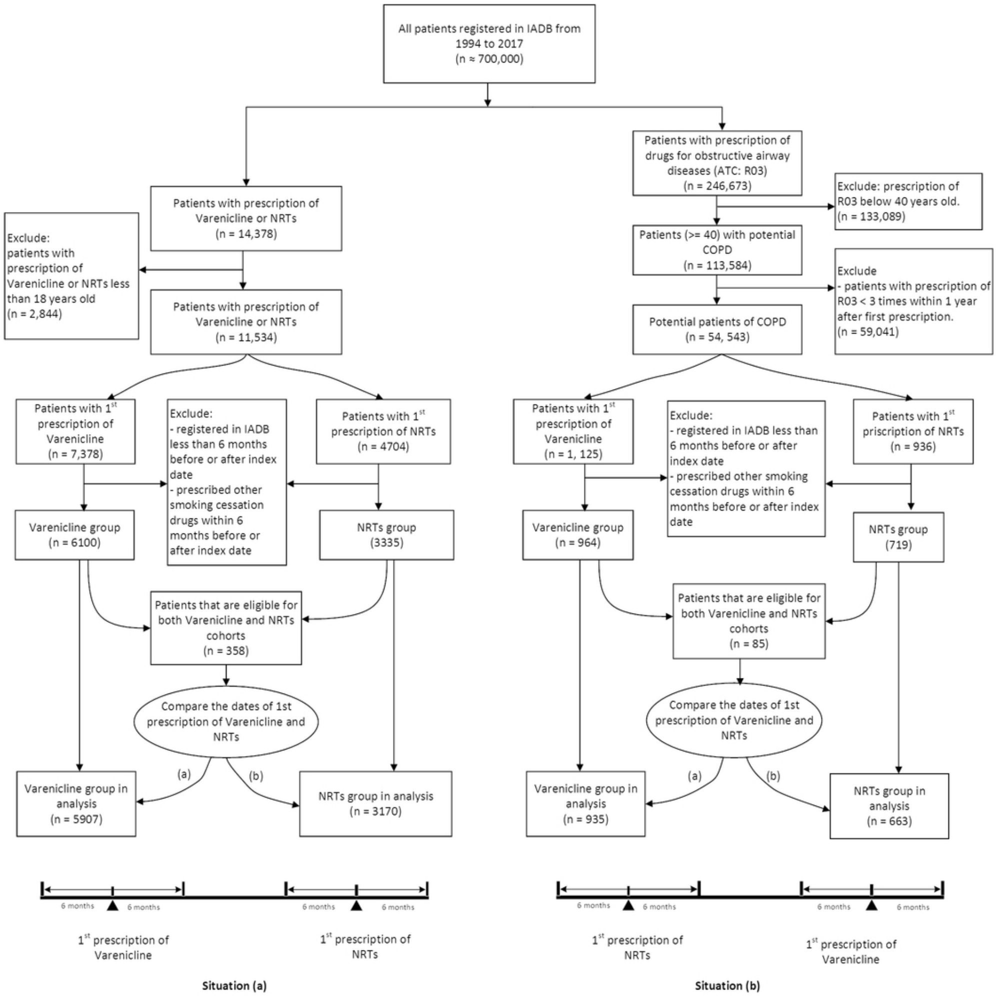

Figure 1. Flow chart of population selection. NRT: nicotine replacement therapies; ATC: anatomical therapeutic chemical.

90x90mm (300 x 300 DPI)

Supplementary Materials

Table S1. List of ATC codes used for identification of related diseases and outcome events.

Variables	ATC codes*	Explanation
COPD	R03	- drugs for obstructive airway diseases;
General psychiatric disorders	N02, N03, N04, N05, N06	- analgesics;<br>- antiepileptics;<br>- anti-Parkinson drugs;<br>- psycholeptics;<br>- psychoanaleptics;
Depression	N06A, N06CA,	- antidepressants;<br>- antidepressants in combination with psycholeptics;
Anxiety	N05B	- anxiolytics;
Insomnia	N05C	- hypnotics and sedatives
Heart failure	C01AA, C03C	- digitalis glycosides<br>- high-ceiling diuretics
Ischemic heart disease	C01DA	- organic nitrates
Hypertension	C02, C03 (except C03C), C07, C08, C09	- antihypertensives<br>- diuretics (except high-ceiling diuretics)<br>- beta blocking agents<br>- calcium channel blockers<br>- agents acting on the renin-angiotensin system
Cancers	L01	- antineoplastic agents
Diabetes mellitus	A10	- drugs used in diabetes
Osteoporosis	M05B	- drugs affecting bone structure and mineralization
Peptic ulcer and GERD	A02B	- drugs for peptic ulcer and gastro-oesophageal reflux disease (gord)
Rheumatic arthritis	M01, M02	- antiinflammatory and antirheumatic products<br>- topical products for joint and muscular pain

For peer review only

Thyroid disorders	H03	- thyroid therapy
Anemia	B03	- antianemic preparations
Glaucoma	S01E	- antiglaucoma preparations and miotics
Gout	M04	- antigout preparations
Allergic rhinitis	R01AD	- corticosteroids

ATC: anatomical therapeutic chemical; COPD: chronic obstructive pulmonary disease; GERD: gastroesophageal reflux disease; *The very specific medication lists under each ATC codes level could be checked in WHO ATC system: https://www.whocc.no/atc_ddd_index/.

For peer review only

Table S2. Subgroup analysis: incidence of neuropsychiatric adverse events (NPAEs) and association with varenicline compared with NRT within follow up of 24 weeks stratified by age and gender.

Subgroups	General populations (N=9077)				COPD populations (N=1958)			
	Psychiatric cohort Varenicline(n=1427) vs NRT(1200)		Non-psychiatric cohort Varenicline(n=4480) vs NRT(n=1970)		Psychiatric cohort Varenicline(n=327) vs NRT(322)		Non-psychiatric cohort Varenicline(n=608) vs NRT(n=341)	
	Crude OR(95% CI)	aOR (95% CI)	Crude OR (95% CI)	aOR (95% CI)	Crude OR (95% CI)	aOR (95% CI)	Crude OR (95% CI)	aOR (95% CI)
Age **								
Age < 40	0.58 [0.32, 1.05]	0.54 [0.28, 1.04]	1.01 [0.61, 1.66]	0.93 [0.54, 1.64]	---	---	---	---
40-65	0.81 [0.65, 1.01]	0.78 [0.63, 0.98]	0.86 [0.71, 1.04]	0.87 [0.72, 1.07]	0.88 [0.56, 1.38]	0.87 [0.54, 1.41]	0.91 [0.57, 1.46]	0.93 [0.57, 1.53]
>65	1.02 [0.66, 1.59]	1.13 [0.70, 1.83]	0.50 [0.33, 0.78]	0.53 [0.34, 0.83]	1.08 [0.50, 2.35]	1.02 [0.43, 2.40]	0.51 [0.25, 1.05]	0.51 [0.24, 1.11]
Gender (n, %) **								
Men	0.87 [0.67, 1.13]	0.89 [0.68, 1.16]	0.71 [0.56, 0.91]	0.78 [0.61, 0.99]	0.95 [0.54, 1.69]	1.24 [0.66, 2.33]	0.81 [0.46, 1.43]	1.00 [0.53, 1.88]
Female	0.73 [0.56, 0.95]	0.74 [0.57, 0.97]	0.89 [0.71, 1.11]	0.91 [0.72, 1.14]	0.78 [0.46, 1.33]	0.77 [0.44, 1.35]	0.73 [0.44, 1.22]	0.76 [0.44, 1.31]

NPAEs: neuropsychiatric adverse events; NRTs: nicotine replacement therapy; OR: odds ratio; CI: confidence interval; aOR: adjusted odds ratio;*adjusted gender, social economic status and comorbidities; **adjusted age, social economic status, comorbidities.

For peer review only

Table S3. Sensitivity analysis: incidence of neuropsychiatric adverse events (NPAEs) and association with varenicline compared with NRT in general population with and without psychiatric disorders within follow up of 12 weeks.

NPAEs	Psychiatric cohort (N=2627) Varenicline (n=1427) vs NRTs (n=1200)			Non-psychiatric cohort (N=6450) Varenicline (n=4480) vs NRTs (n=1970)		
	Events (n, %)*	Crude OR (95% CI)	Adjusted OR# (95% CI)	Events (n, %)*	Crude OR (95% CI)	Adjusted OR# (95% CI)
**Follow-up of 12 weeks**						
Overall	1062 (74.4): 948 (79.0)	0.77 [0.64, 0.93]	0.78 [0.64, 0.94]	363 (8.1): 223 (11.3)	0.69 [0.58,0.82]	0.74 [0.62, 0.89]
Depression	560 (39.2): 491 (40.9)	0.93[0.80, 1.09]	0.89 [0.76, 1.05]	86 (1.9): 30 (1.5)	1.27 [0.83, 1.92]	1.29 [0.84, 1.97]
Anxiety	367 (25.7): 423 (35.3)	0.64 [0.54, 0.75]	0.63 [0.53, 0.74]	119 (2.7): 61 (3.1)	0.85 (0.62, 1.17)	0.90 [0.65, 1.24]
Insomnia	321 (22.5): 311 (25.9)	0.83 [0.69, 0.99]	0.88 [0.73, 1.05]	82 (1.8): 61 (3.1)	0.58 [0.42, 0.82]	0.62 [0.44, 0.88]

NPAE: neuropsychiatric adverse events; OR: odds ratio; CI: confidence interval; NRTs: nicotine replacement therapy; # adjusted for age, gender, social economic status and related comorbidities. *Varenicline:NRT.

For peer review only

Table S4. Sensitivity analysis: incidence of neuropsychiatric adverse events (NPAEs) and association with compared with NRT in general population within follow up of 24 weeks.

Limitations	General populations (N=9077)					
	Psychiatric cohort varenicline(n=1427) vs NRTs (n=1200)			Non-psychiatric cohort varenicline(n=4480) vs NRTs (n=1970)		
	Events (n, %) #	OR (95% CI)	aOR (95% CI) *	Events (n, %) #	OR (95% CI)	aOR (95% CI) *
**Exclude participants prescribed drugs for any NSP within 1 month before index date**						
Overall	254 (57.6): 157 (60.4)	0.89 [0.65, 1.22]	0.87 [0.63, 1.21]	418 (9.6): 192 (10.4)	0.91 [0.76, 1.10]	0.93 [0.78, 1.12]
Depression	158 (35.8): 82 (31.5)	1.21 [0.88, 1.68]	1.18 [0.84, 1.67]	139 (3.2): 50 (2.7)	1.18 [0.85, 1.64]	1.16 [0.84, 1.62]
Anxiety	78 (17.7): 62 (23.8)	0.69 [0.47, 0.10]	0.68 [0.46, 1.01]	197 (4.5): 85 (4.6)	0.98 [0.76, 1.27]	1.02 [0.78, 1.33]
Insomina	75 (17.0): 48 (18.5)	0.94 [0.74, 1.20]	0.94 [0.62, 1.43]	131 (3.0): 91 (4.9)	0.60 [0.46, 0.79]	0.61 [0.46, 0.80]
**Exclude participants prescribed drugs for depression, anxiety and insomnia within 1 month before index date**						
Overall	314 (48.9): 201 (46.1)	1.12 [0.88, 1.43]	1.05 [0.82, 1.36]	424 (9.6): 203 (10.6)	0.90 [0.75, 1.07]	0.92 [0.77, 1.11]
Depression	191 (29.8): 104 (23.9)	1.35 [1.02, 1.79]	1.25 [0.93, 1.66]	143 (3.3): 52 (2.7)	1.20 [0.87, 1.66]	1.19 [0.86, 1.65]
Anxiety	99 (15.4): 81 (18.6)	0.80 [0.58, 1.10]	0.75 [0.54, 1.05]	199 (4.5): 92 (4.8)	0.94 [0.73, 1.21]	0.98 [0.75, 1.26]
Insomnia	92 (14.3): 63 (14.4)	0.99 [0.70, 1.40]	1.03 [0.72, 1.47]	132 (3.0): 94 (4.9)	0.60 [0.46, 0.78]	0.61 [0.47, 0.81]
**Exclude participants whose study period includes policies changes**						
Overall	797 (76.0): 733 (77.7)	0.91 [0.74, 1.12]	0.86 [0.69, 1.07]	352 (11.1): 205 (12.9)	0.85 [0.70, 1.02]	0.86 [0.71, 1.03]
Depression	468 (44.7): 411 (43.6)	1.04 [0.88, 1.25]	0.98 [0.82, 1.18]	115 (3.6): 45 (2.8)	1.30 [0.91, 1.84]	1.28 [0.90, 1.82]
Anxiety	343 (32.7): 372 (39.4)	0.75 [0.62, 0.90]	0.72 [0.60, 0.87]	169 (5.3): 95 (6.0)	0.89 [0.69, 1.15]	0.91 [0.70, 1.19]
Insomnia	273 (26.0): 280 (29.7)	0.83 [0.69, 1.02]	0.87 [0.71, 1.07]	109 (3.4): 83 (5.2)	0.65 [0.48, 0.87]	0.66 [0.49, 0.88]
**Exclude selection bias for treatments by applying the inverse probability weighted regression**						
Overall	1074 (75.3): 942 (78.5)	0.83 [0.69, 1.00]	0.88 [0.77, 1.00]	469 (10.5): 248 (12.6)	0.81 [0.69, 0.96]	0.89 [0.80, 0.99]

For peer review only

Depression	629 (44.1): 548 (45.7)	0.94 [0.80, 1.09]	0.90 [0.81,1.00]	148 (3.3): 57 (2.9)	1.15 [0.84, 1.56]	1.14 [0.94, 1.39]
Anxiety	456 (32.0): 472 (39.3)	0.72 [0.62, 0.85]	0.71 [0.63, 0.80]	215 (4.8): 110 (5.6)	0.85 [0.67, 1.08]	0.92 [0.78, 1.07]
Insomina	372 (26.1): 352 (29.3)	0.85 [0.72, 1.01]	0.89 [0.79, 1.00]	147 (3.3): 105 (5.3)	0.60 [0.47, 0.78]	0.63 [0.53, 0.75]

NPAEs: neuropsychiatric adverse events; NRTs: nicotine replacement therapy; OR: odds ratio; aOR: adjusted odds ratio; CI: confidence interval; *adjusted age, gender, social economic status and comorbidities. We set the indexdate was within period between July 1st, 2011 and June 30, 2013; Policy change: In the Netherlands, pharmacologic Smoking Cessation Treatments (pSCTs) were reimbursed in 2011. In 2012 the reimbursement was discontinued. As of 2013, pSCTs were again reimbursed, provided they are accompanied by behavioural counselling. #Varenicline: NRT.

For peer review only

Table S5. Sensitivity analysis: incidence of neuropsychiatric adverse events (NPAEs) and association with varenicline compared with NRT in COPD population with and without psychiatric disorders within follow up of 12 weeks.

Outcomes	**Psychiatric cohort (N=649)** Varenicline (n=327) vs NRTs (n=322)			**Non-psychiatric cohort (N=949)** Varenicline (n=608) vs NRTs (n=341)		
	Events (n, %)[#]	Crude OR (95% CI)	Adjusted OR* (95% CI)	Events (n, %)[#]	Crude OR (95% CI)	Adjusted OR* (95% CI)
**Follow-up of 12 weeks**						
Overall	260 (79.5): 267 (82.9)	0.80 [0.54, 1.19]	0.89 [0.59, 1.36]	63 (10.4): 45 (13.2)	0.76 [0.51, 1.14]	0.84 [0.54, 1.29]
Depression	134 (41.0): 127 (39.4)	1.07 [0.78, 1.46]	0.94 [0.67, 1.32]	13 (2.1): 5 (1.5)	1.47 [0.52, 4.15]	1.95 [0.65, 5.91]
Anxiety	95 (29.1): 123 (38.2)	0.66 [0.48, 0.92]	0.64 [0.45, 0.90]	16 (2.6): 13 (3.8)	0.68 [0.32, 1.44]	0.68 [0.31, 1.52]
Insomnia	88 (26.9): 114 (35.4)	0.67 [0.48, 0.94]	0.72 [0.51, 1.03]	20 (3.3): 11 (3.2)	1.02 [0.48, 2.16]	1.12 [0.51, 2.46]

NPAE: neuropsychiatric adverse events; OR: odds ratio; CI: confidence interval; NRTs: nicotine replacement therapy; *adjusted for age, gender, social economic status and related comorbidities. [#]Varenicline:NRT.

For peer review only

Table S6. Sensitivity analysis: incidence of neuropsychiatric adverse events (NPAEs) and association with compared with NRT in COPD population within follow up of 24 weeks.

Limitations	COPD populations					
	Psychiatric cohort (N=649) varenicline(n=327) vs NRTs(322)			Non-psychiatric cohort (N=949) varenicline(608) vs NRTs(341)		
	Events (n, %)[#]	OR (95% CI)	aOR (95% CI)[*]	Events (n, %)[#]	OR (95% CI)	aOR (95% CI)[*]
**Exclude participants prescribed drugs for any NSP within 1 month before index date**						
Overall	54 (60.0): 39 (61.9)	0.92 [0.48, 1.79]	0.85 [0.41, 1.77]	72 (12.1): 40 (12.7)	0.95 [0.63, 1.43]	0.92 [0.60, 1.43]
Depression	29 (32.2): 19 (30.2)	1.10 [0.55, 2.21]	0.88. [0.39, 1.97]	20 (3.4): 10 (3.2)	1.06 [0.49, 2.29]	1.12 [0.50, 2.52]
Anxiety	22 (24.4): 20 (31.7)	0.70 [0.34, 1.42]	0.87 [0.40, 1.90]	38 (6.4): 19 (6.0)	1.06 [0.60, 1.87]	1.10 [0.60, 2.01]
Insomina	15 (16.7): 16 (25.4)	0.59 [0.27, 1.30]	0.60 [0.24, 1.49]	25 (4.2): 16 (5.1)	0.82 [0.43, 1.56]	0.71 [0.36, 1.37]
**Exclude participants prescribed drugs for depression, anxiety and insomnia within 1 month before index date**						
Overall	68 (51.1): 48 (47.5)	1.16 [0.69, 1.94]	1.29 [0.73, 2.26]	73 (12.1): 43 (13.0)	0.92 [0.62, 1.38]	0.93 [0.61, 1.42]
Depression	35 (26.3): 23 (22.8)	1.21 [0.66, 2.22]	1.08 [0.56, 2.08]	21 (3.5): 10 (3.0)	1.16 [0.54, 2.49]	1.33 [0.60, 2.99]
Anxiety	27 (20.3): 23 (22.8)	0.86 [0.46, 1.62]	0.97 [0.49, 1.91]	38 (6.3): 21 (6.4)	0.99 [0.57, 1.72]	1.00 [0.56, 1.79]
Insomnia	21 (15.8): 20 (19.8)	0.76 [0.39, 1.49]	0.87 [0.42, 1.82]	25 (4.2): 17 (5.2)	0.80 [0.43, 1.50]	0.72 [0.37, 1.39]
**Exclude participants whose study period includes policies changes**						
Overall	203 (78.1): 211 (80.2)	0.88 [0.58, 1.34]	0.97 [0.62, 1.51]	63 (14.2): 41 (15.1)	0.93 [0.61, 1.43]	1.00 [0.64, 1.58]
Depression	114 (43.8): 110 (41.8)	1.09 [0.77, 1.54]	0.96 [0.66, 1.40]	18 (4.1): 7 (2.6)	1.60 [0.66, 3.89]	1.73 [0.68, 4.42]
Anxiety	89 (34.2): 109 (41.4)	0.74 [0.52, 1.05]	0.72 [0.49, 1.05]	35 (7.9): 20 (7.4)	1.08 [0.61, 1.91]	1.21 [0.66, 2.24]
Insomnia	81 (31.2): 106 (40.3)	0.67 [0.47, 0.96]	0.75 [0.51, 1.10]	19 (4.3): 15 (5.5)	0.77 [0.38, 0.53]	0.71 [0.34, 1.45]
**Exclude selection bias for treatments by applying the inverse probability weighted regression**						
Overall	258 (78.9): 260 (80.7)	0.89 [0.61, 1.31]	0.99 [0.76, 1.30]	76 (12.5): 53 (15.5)	0.78 [0.53, 1.13]	0.82 [0.63, 1.07]

For peer review only

Depression	146 (44.6): 145 (45.0)	0.99 [0.72, 1.34]	0.90 [0.73, 1.12]	22 (3.6): 10 (2.9)	1.24 [0.58, 2.66]	1.39 [0.84, 2.29]
Anxiety	110 (33.6): 136 (42.2)	0.69 [0.50, 0.95]	0.67 [0.54, 0.84]	38 (6.3): 25 (7.3)	0.84 [0.50, 1.42]	0.87 [0.60, 1.25]
Insomina	102 (31.2): 124 (38.5)	0.72 [0.52, 1.00]	0.86 [0.68, 1.08]	25 (4.1): 21 (6.2)	0.65 [0.36, 1.19]	0.62 [0.41, 0.94]

NPAEs: neuropsychiatric adverse events; NRTs: nicotine replacement therapy; OR: odds ratio; *aOR: adjusted odds ratio; *adjusted age, gender, social economic status and comorbidities. # Varenicline: NRT. We set the indexdate was within period between July 1st, 2011 and June 30, 2013; Policy change: In the Netherlands, pharmacologic Smoking Cessation Treatments (pSCTs) were reimbursed in 2011. In 2012 the reimbursement was discontinued. As of 2013, pSCTs were again reimbursed, provided they are accompanied by behavioural counselling.

For peer review only

STROBE Statement—Checklist of items that should be included in reports of cohort studies

	Item No	Recommendation	Page No
**Title and abstract**	1	(*a*) Indicate the study's design with a commonly used term in the title or the abstract	1
		(*b*) Provide in the abstract an informative and balanced summary of what was done and what was found	2
**Introduction**			
Background/rationale	2	Explain the scientific background and rationale for the investigation being reported	4
Objectives	3	State specific objectives, including any prespecified hypotheses	5
**Methods**			
Study design	4	Present key elements of study design early in the paper	5
Setting	5	Describe the setting, locations, and relevant dates, including periods of recruitment, exposure, follow-up, and data collection	5,6,7
Participants	6	(*a*) Give the eligibility criteria, and the sources and methods of selection of participants. Describe methods of follow-up	5,6
		(*b*) For matched studies, give matching criteria and number of exposed and unexposed	N/A
Variables	7	Clearly define all outcomes, exposures, predictors, potential confounders, and effect modifiers. Give diagnostic criteria, if applicable	6, 7
Data sources/ measurement	8*	For each variable of interest, give sources of data and details of methods of assessment (measurement). Describe comparability of assessment methods if there is more than one group	5, 6, 7
Bias	9	Describe any efforts to address potential sources of bias	6, 7
Study size	10	Explain how the study size was arrived at	N/A
Quantitative variables	11	Explain how quantitative variables were handled in the analyses. If applicable, describe which groupings were chosen and why	7
Statistical methods	12	(*a*) Describe all statistical methods, including those used to control for confounding	6,7,8
		(*b*) Describe any methods used to examine subgroups and interactions	6,7
		(*c*) Explain how missing data were addressed	N/A
		(*d*) If applicable, explain how loss to follow-up was addressed	N/A
		(*e*) Describe any sensitivity analyses	7
**Results**			
Participants	13*	(a) Report numbers of individuals at each stage of study—eg numbers potentially eligible, examined for eligibility, confirmed eligible, included in the study, completing follow-up, and analysed	8
		(b) Give reasons for non-participation at each stage	8
		(c) Consider use of a flow diagram	20
Descriptive data	14*	(a) Give characteristics of study participants (eg demographic, clinical, social) and information on exposures and potential confounders	8
		(b) Indicate number of participants with missing data for each variable of interest	N/A
		(c) Summarise follow-up time (eg, average and total amount)	8,9
Outcome data	15*	Report numbers of outcome events or summary measures over time	8, 9,10, 20
**Discussion**			

For peer review only

Key results	18	Summarise key results with reference to study objectives	10,11,12
Limitations	19	Discuss limitations of the study, taking into account sources of potential bias or imprecision. Discuss both direction and magnitude of any potential bias	13, 14
Interpretation	20	Give a cautious overall interpretation of results considering objectives, limitations, multiplicity of analyses, results from similar studies, and other relevant evidence	10,11,12, 13
Generalisability	21	Discuss the generalisability (external validity) of the study results	13
**Other information**			
Funding	22	Give the source of funding and the role of the funders for the present study and, if applicable, for the original study on which the present article is based	15

*Give information separately for exposed and unexposed groups.

Note: An Explanation and Elaboration article discusses each checklist item and gives methodological background and published examples of transparent reporting. The STROBE checklist is best used in conjunction with this article (freely available on the Web sites of PLoS Medicine at http://www.plosmedicine.org/, Annals of Internal Medicine at http://www.annals.org/, and Epidemiology at http://www.epidem.com/). Information on the STROBE Initiative is available at http://www.strobe-statement.org.

For peer review only
